# Dissecting conjugation and electronic effects on the linear and non-linear optical properties of rhenium(i) carbonyl complexes†

**DOI:** 10.1039/d2cp03844g

**Published:** 2022-10-31

**Authors:** Ricardo J. Fernández-Terán, Estefanía Sucre-Rosales, Lorenzo Echevarria, Florencio E. Hernández

**Affiliations:** Department of Chemistry, University of Sheffield Sheffield S3 7HF UK Ricardo.Fernandez@sheffield.ac.uk Ricardo.FernandezTeran@gmail.com; Department of Chemistry, University of Zurich Winterthurerstrasse 190 CH-8057 Zurich Switzerland; Department of Chemistry, University of Central Florida Orlando Florida 32816 USA; Departamento de Química, Universidad Simón Bolívar Caracas 1080-A AP 89000 Venezuela; CREOL/The College of Optics and Photonics, University of Central Florida Orlando Florida 32816 USA

## Abstract

Herein, we report a theoretical and experimental analysis of the conjugation and electronic effects on the one-photon (1PA) and two-photon absorption (2PA) properties of a series of Re(i) carbonyl complexes with terpyridine-based ligands. An excellent agreement was obtained between the calculated and experimental 2PA spectra of the *κ*^2^*N*-terpyridine tricarbonyl complexes (1a-b), with 2PA cross sections reaching up to *ca.* 40 GM in DMF. By stepwise lowering the conjugation length in the terpy ligand and changing the local symmetry around the metal centre, we show that conjugation and delocalisation play a major role in increasing 2PA cross sections, and that the character of the excited states does not directly enhance the non-linear properties of these complexes—contrary to the results observed in 1PA. Altogether, these results give valuable guidelines towards more efficient two-photon-absorbing coordination complexes of Re(i), with potential applications in photodynamic therapy and two-photon imaging.

## Abstract 2

In our work, we correlate the observed computational and experimental trends in one- and two-photon absorption cross sections with structural parameters in Re(i) carbonyl complexes, providing design criteria towards useful two-photon photosensitisers.

## Graphical abstract

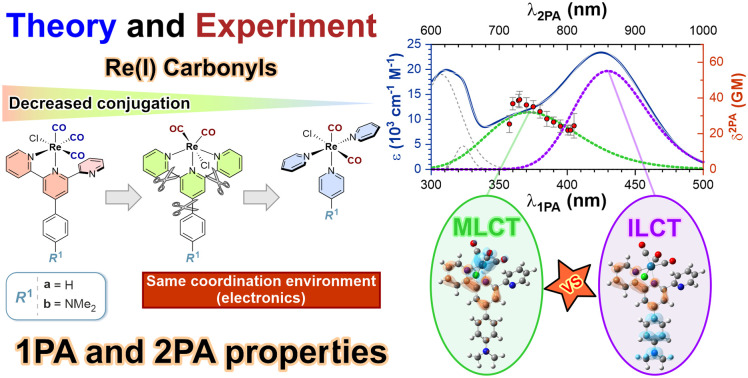

## I. Introduction

The synthesis, characterisation and spectroscopic study of rhenium(i) carbonyl complexes bearing diimine ligands has been an active field of research in the last few decades. Since their introduction by Wrighton and co-workers,^1–3^ and pioneering work by Lehn and co-workers,^4^ they have gained enormous popularity thanks to their widespread use as CO_2_ reduction catalysts,^5–11^ and photosensitisers.^12–23^

**Scheme 1:**
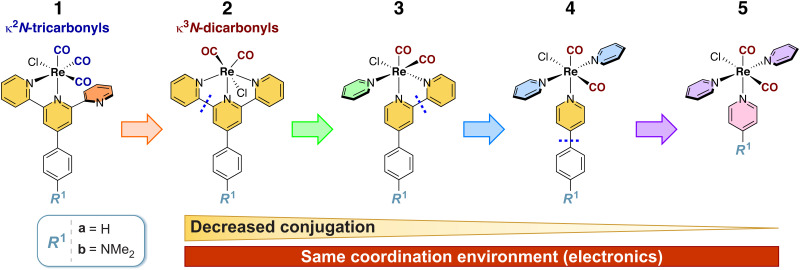
Structures of the complexes studied in this work. The blue dashed segments show which bond(s) are broken when decreasing the conjugation stepwise from 2 to 5. See the text for discussion.

In our previous work, some of us have reported on the photophysics and photochemistry of different rhenium(i) *κ*^2^*N*-tricarbonyl (Scheme 1, 1),^24^ and *κ*^3^*N*-dicarbonyl terpyridine complexes (Scheme 1, 2),^25^ based on a 4′-(4-substituted-phenyl)-2,2′:6′,2′′-terpyridine ligand. The groups of Castellano,^26^ Sullivan,^27^ Dempsey,^28^ Ishitani,^29^ and others^30–32^ have studied different rhenium(i) carbonyl complexes with diimine ligands, in terms of their ground and excited-state properties.

A constant challenge in the design of these complexes is the extension of their absorption to the visible and near-infrared (NIR) region of the electromagnetic spectrum. We have shown that by introducing a strongly donating group like NMe_2_ in a remote position of the ligand framework, there is a change in the excited-state character (*e.g.* between complexes 1a and 1b, Scheme 1) from metal-to-ligand charge transfer (MLCT) to intraligand charge transfer (ILCT). This resulted in a red shift of *ca.* 100 nm in the absorption maximum and a ∼200-fold increase in the lifetime, accompanied by a ∼5-fold increase in the extinction coefficient.^24^

In contrast, the analogous *κ*^3^*N*-dicarbonyl terpyridine complexes (2a-b) have an excited state of MLCT character irrespective of the substituent in a remote position, as shown by transient IR (TRIR), and are non-emissive.^25^ Hanan and co-workers have shown that the replacement of the axial halide ligand in similar complexes leads to the recovery of NIR emission.^33^ More recently, Machura and co-workers have studied Re(i) carbonyl complexes of different terpyridine-type ligands with appended functionalities, the role of solvent polarity and the triimine core in the ground- and excited-state properties of these complexes.^34–37^

Gordon and co-workers have recently reported on a series of tricarbonyl Re(i) complexes with thiophene-based donor–acceptor systems with varying degrees of ILCT and localised π,π* characters.^38^ They have also reported on a series of Pt(ii) bis(acetylide) and Re(i) tricarbonyl complexes with triphenylamine-substituted phenanthroline ligands, in which the MLCT and ILCT characters were switched as a function of substituents on the triphenylamine group.^39^ Further reports from the Gordon group on [Ru(N∩N)_3_]^2+^ complexes with N∩N ligands derived from substituted 1,10-phenanthroline showed that appending aromatic groups to the ligand changes the character from MLCT to a ligand-centred triplet (^3^LC).^40^

Effectively controlling the character of the lowest excited states of these complexes thus provides a unique opportunity towards tuning their optical properties, whilst maintaining (and even increasing) their excited-state lifetimes—a crucial aspect to optimise, if these complexes are to find applications in photocatalysis.

Whilst most studies focus on the linear absorption and emission properties, the non-linear optical properties of these complexes—in particular their two-photon absorption (2PA) cross sections—have not been studied to the same extent. Most complexes showing 2PA have been derived from two-photon absorbing ligands decorated with a metal centre,^41^ as it has been shown that the latter can have a significant impact on the 2PA cross section.^42^ These results show the importance of conjugation in these D–A systems (where the terpyridine moiety acts as an electron acceptor of the electron density of the metal upon complexation), and provides the possibility of ILCT and MLCT transitions. Complexes of Zn(ii), Cd(ii), Mn(ii), Pt(ii), and Ru(ii) with terpyridine-based ligands have been studied in the past, with the closed-shell d^10^ complexes showing the highest 2PA cross sections overall.^43,44^ The 2PA properties of some Re(i) carbonyl complexes have been studied,^45^ but no significant structure–property relationships or 2PA cross-sections in Re(i) carbonyl complexes have been reported to date: most of the attention in this regard has been focused on Ru(ii) polypyridyl^46^ and cyclometallated Ir(iii) complexes.^47,48^

Two-photon absorption provides an additional pathway for efficient energy conversion, allowing access to electronically excited states across a greater portion of the spectrum. This could, for example, lead to the production of reactive oxygen species by sensitisation of oxygen with NIR irradiation, an approach often used in photodynamic therapy (PDT). In addition, 2PA processes lead to a significantly higher spatial resolution (since high intensity is required for 2PA to take place). Hence, 2PA dyes could lead to localised imaging or PDT with transition metal complexes, as has been previously demonstrated with Ru(ii) and Ir(iii) complexes.^46–51^

Herein, we present a hybrid experimental and computational study of a series of Re(i) carbonyl complexes, aiming to elucidate the relationship between their structure and their photophysical, photochemical and non-linear optical properties.

Considering 1a-b and 2a-b as starting points, we investigated computationally using density functional theory (DFT) three additional variants of the parent complexes (3–5, Scheme 1) with decreasing conjugation, but similar ligand environments around the metal centre: 3a-b (*cis*-[Re(*κ*^2^*N*-4-*R*^1^-phenyl-2,2′-bipyridine)(py)(CO)_2_Cl]); 4a-b (*trans*,*cis*-[Re(*κ*^1^*N*-4-(4-*R*^1^-phenyl)pyridine)(py)_2_(CO)_2_Cl]); and 5a-b, (*trans*,*cis*-[Re(4-*R*^1^-py)(py)_2_(CO)_2_Cl]). In all cases, *R*^1^ = H (a) and *R*^1^ = NMe_2_ (b).

These complexes result from stepwise lowering of the conjugation length in the terpy ligand (by breaking apart the constituent pyridine rings), accompanied by changes in the local symmetry around the Re(i) centre. Complexes 3a-b share the canonical structure of 1a-b and the *κ*^2^*N* coordination motif, whilst retaining *cis*-dicarbonyls and having three coordinated pyridines (like 2a-b), hence representing a midpoint between the two series. Complexes 4a-b and 5a-b represent a stepwise decrease in the number of conjugated rings, with complex 5a having only pyridines as ligands, and complex 5b having a NMe_2_ group directly linked to one of the pyridine ligands. Other authors have considered directly substituted 4′-*R*^1^-2.2′:6′,2′′-terpyridine-based ligands (and their Ru(ii) complexes, which are good candidates for photodynamic therapy).^52^ We, however, focus on a remote substitution strategy to develop strongly-absorbing and long-lived complexes in a complementary manner.

This approach allowed us to elucidate the contribution of conjugation and different electronic and geometric effects in the linear (one-photon absorption, 1PA) and non-linear (two-photon absorption, 2PA) optical properties of these complexes, providing valuable design criteria towards useful two-photon absorbing dyes based on Re(i) carbonyls.

## II. Materials and methods

### A Synthesis and spectroscopic characterisation

Solvents used for synthesis were of reagent grade or higher, whilst those for spectroscopic measurements were of HPLC or spectroscopic grades and were used as received. Complexes 1a-b,^24^ and 2a-b^25^ were synthesised according to previously published methods. UV-Vis absorption spectra were recorded on a Shimadzu UV-3600 Plus absorption spectrometer using standard 1 × 1 cm pathlength quartz cuvettes.

### B Experimental determination of the degenerate non-linear (two-photon) absorption cross sections

An open-aperture single-beam Z-scan setup was used to determine the degenerate 2PA cross sections. In brief, an ultrafast Ti:Sapphire amplifier (Coherent Legend) producing *ca.* 90 fs pulses centred at 800 nm was used to pump an optical parametric amplifier (Coherent OPerA Solo), which provided a tunable excitation beam in the 730–900 nm region (typical FWHM of 10 nm). The output of the OPA was spatially filtered with a 100 μm pinhole at the centre of a 1:1 telescope to ensure a Gaussian intensity profile, then split in two branches (sample and reference beams, respectively). The sample beam was passed through a Glan–Taylor polarising cube, then focused using a fused silica lens into a 1 mm pathlength quartz cuvette containing the sample solution, which was scanned along the focal axis using a computer-controlled stage. After passing the solution, the sample beam was refocused into a single-element photodiode. The reference beam was focused into a second identical detector, and was used to account for shot-to-shot fluctuations, as described in ref. 53. The normalised transmittance was fitted according to eqn (1),^53,54^ with the 2PA cross section (*δ*^2PA^) and the beam waist [*ω*_0_, where *z*_*R*_ = π*ω*_0_^2^/(*M*^2^*λ*)] as free fit parameters.1
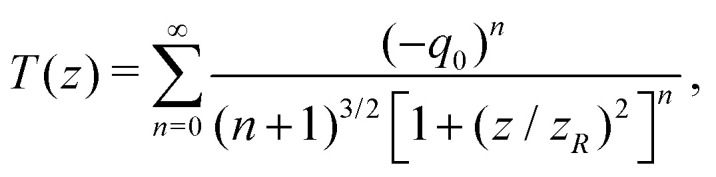


In this equation, *q*_0_ = *βI*_0_*L*_eff_, and *L*_eff_ ≈1 mm (dilute limit). The two-photon absorption coefficient (*β*) is related to the two-photon absorption cross section (*δ*^2PA^, in cm^4^ s photon^−1^ molecule^−1^) by eqn (2), where *C* is the concentration of the absorbing species (in mol L^−1^), *N*_A_ is Avogadro's constant, *ħ* is the reduced Planck constant and *ω* is the photon frequency:2
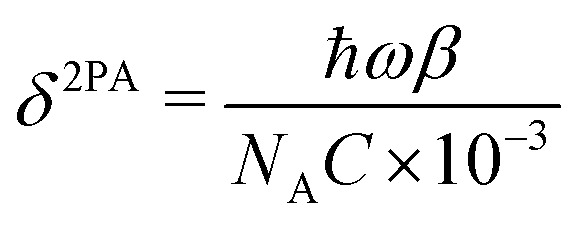


The beam waist (*ω*_0_^2^) was experimentally determined by fitting a two-dimensional Gaussian function to the image profile of an attenuated beam obtained using a CMOS sensor (OmniVision OV5648, 5 MP sensor). The so-obtained values were used as initial guesses to fit eqn (1) to the experimental data, and the values agreed in all cases within experimental error. For the fitting procedure, eqn (1) was truncated after the 10th term.

The peak intensity (*I*_0_) was calculated from the experimentally determined pulse temporal FWHM (*τ*), repetition rate (*R*), beam waist (*ω*_0_^2^, assuming *M*^2^ = 1.3 from the laser specifications) and average power (*P*_avg_), as follows eqn (3):^54^3
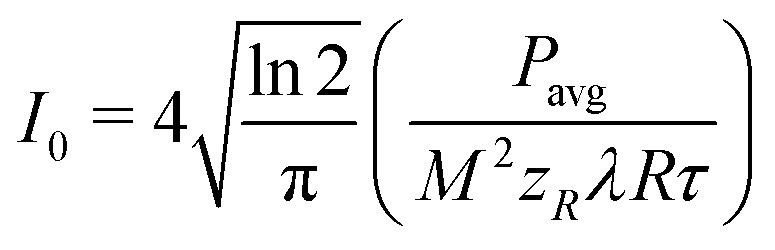


Solutions of the corresponding complexes were freshly prepared in DMF of spectroscopic grade (Sigma-Aldrich) at a concentration of not more than 5 μm (often limited by the solubility of the complexes in DMF at room temperature). The repetition rate of the laser was kept at 50 Hz (using the laser timing electronics) to avoid contributions from thermal effects, and the pulse energies at the sample position were kept below 0.4 μJ.

### C Geometry optimisations and calculated UV-Vis (linear) absorption spectra

Geometry optimisations were performed using the Gaussian 16 rev. B.01 software.^55^ The geometry from the crystal structures of 1a^56^ and 2b^25^ were used as initial guesses for their corresponding series, and were then optimised with tight convergence criteria using the 6-311G(d,p) basis set for all light atoms, and the LANL2DZ effective core potential (ECP) and basis set for Re,^57–59^ with the hybrid B3LYP^60–62^ functional.

The IEF-PCM solvation model (as implemented in Gaussian) was used for calculations in *N*,*N*-dimethylformamide (DMF) solution.^63^ The structures of all other complexes, and of the terpyridine ligands associated with complexes 1a-b and 2a-b (hence denoted La and Lb, respectively) were also optimised at this level of theory, and these converged geometries were used in all further calculations. Harmonic vibrational analysis revealed no negative frequencies, confirming the structures to be true minima.

Time-dependent density functional theory (TD-DFT) was performed on the respective ground-state optimised geometries. For the complexes, we considered the lowest 30 singlets and 30 triplets [using the td = (nstates = 30,50-50) option in Gaussian], whilst for the ligands only the lowest 30 singlets were considered.

The calculated UV-Vis absorption spectra were obtained by convolution with a Gaussian line shape function with a uniform full width at half maximum (FWHM) of 4840 cm^−1^ (0.6 eV). The experimental UV-Vis linear absorption spectra (1PA) of complexes 1a-b and 2a-b were reproduced from our previous work.^24,25^

### D Excited state analysis

Fragment-based population analysis and transition density decomposition analysis were performed using the TheoDORE program (v3.0),^64^ with the inbuilt transition density matrix analysis modules.^65,66^ Calculations considering only the lowest 30 singlet states of all complexes were performed, including the options pop = full iop(9/40 = 3) for compatibility. The energies and oscillator strengths obtained from these calculations were identical to those that included the triplet states. The TheoDORE program internally uses the CCLIB library (v1.7.2)^67^ to parse the output of the Gaussian calculations. Charge density difference isosurfaces (CDD) were generated using the MultiWfn program (v3.7).^68^ Additional figures and details are given in the ESI,† sections 1.7 (fragment-based analysis) and 1.8 (CDDs).

### E Theoretical estimation of the degenerate non-linear (two-photon) absorption cross sections

Degenerate two-photon absorption (2PA) cross sections were calculated using the Dalton program (release 2020.0),^69,70^ and its implementation of the TD-DFT singlet quadratic response,^71,72^ following a similar procedure as in our previous reports.^73,74^

Calculations were performed in the Dalton software with the B3LYP functional (using the B3LYPg option to replicate the Gaussian implementation), and identical basis sets as described above, using the corresponding geometries optimised in Gaussian as described above. The 20 lowest-energy states were calculated for each complex.

The 2PA cross sections and spectra were calculated according to eqn (4–7):^75^4
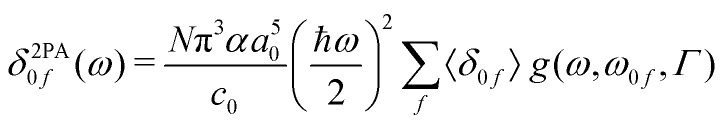
5
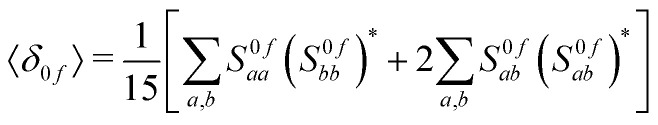
where *c*_0_ = 2.99792458 × 10^10^ cm s^−1^ is the speed of light in vacuum; *a*_0_ = 5.29177210903 × 10^−9^ cm is the Bohr radius; *α* = 0.0072973525693 is the fine structure constant; *E* = *ħω* is the transition energy and *ħω*/2 is the photon energy for the degenerate 2PA case; 〈*δ*_0*f*_〉 is the orientation-averaged degenerate two-photon transition probability for a linearly polarized laser beam; and *Γ* is the half-width half-maximum (HWHM) linewidth. The ground, virtual and final states are represented by 0, *k* and *f*, respectively.

In eqn (5), the subscripts *a*,*b* represent the Cartesian coordinates, where *S*^0*f*^_*a*,*b*_ is the two-photon matrix element, defined as:^76^6
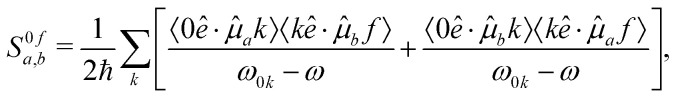
where *μ̂*_*i*_ represents the *i*-th component of the dipole moment, and *ê* is a unit vector along the polarisation direction of the incident light field. A normalised Lorentzian line shape function, *g*(*ω*,*ω*_0*f*_,*Γ*), was used to broaden the calculated responses and obtain the 2PA spectra eqn (7):7
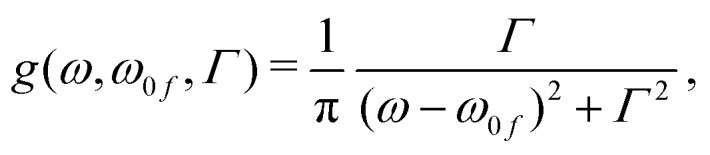
with the constants given before, and if 〈*δ*_0*f*_〉, *ω* and *Γ* are given in atomic units in eqn (4) and (5), the calculated 2PA cross sections can be converted to Göppert Mayer units by multiplying eqn (4) by 10^50^ (since 1 GM = 10^−50^ cm^4^ s photon^−1^). We have used *N* = 4 in eqn (4) for better comparison with single-beam Z-scan measurements, as is nicely discussed in ref. 77. All calculated 2PA spectra were obtained by convolution with a Lorentzian function with *Γ* = 0.2 eV, a vertical scaling factor of 0.75, and a red shift of 0.1 eV for all transitions.

Given the qualitative nature of our discussion of the calculated 2PA cross sections, we ignored the states with unrealistic cross sections [〈*δ*_0*f*_〉 ≥ 10^6^ a.u.], obtained for some complexes. These states may appear due to resonance enhancements—since the denominator in eqn (6) approaches zero in resonance—yielding a value for the cross section that tends to infinity (subject to numerical error and rounding artifacts). These states are somewhat arbitrary in occurrence and are no longer dominated by the electronic structure, other than indirectly, through the separation of the excited states in the molecule. Hence, we focus on the states with physically meaningful cross sections for our theoretical discussion of these complexes and the comparison with experimental values.

## III Results and discussion

### A. Linear absorption spectra

**Fig. 1:**
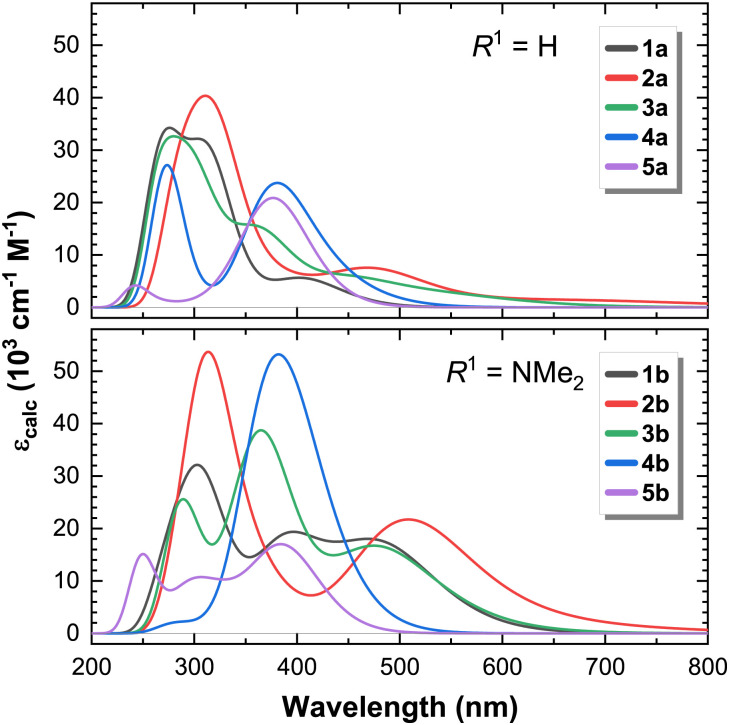
Calculated 1PA spectra of complexes (1–5)a (*R*^1^ = H, top) and (1–5)b (*R*^1^ = NMe_2_, bottom).

We begin our discussion by examining the calculated linear (1PA) absorption spectra (Fig. 1). The calculated UV-Vis absorption spectra show a good agreement with experimental results for compounds 1a-b and 2a-b, previously reported in ref. 24 and 25, respectively. These results validate the level of theory chosen to describe these complexes. The energies and oscillator strengths of all 1PA transitions for all complexes and the ligands La and Lb are provided in the ESI,† Table S1. An overlay of the calculated and experimental spectra of 1a-b and 2a-b is given in Fig. S11 in the ESI.†

Amongst the unsubstituted complexes (a-series), absorption takes place predominantly below 500 nm, except for complexes 2a and 3a. Altogether, these complexes perform relatively poorly as light absorbers. Complexes in the b-series, on the other hand, show notably increased absorption coefficients across the entire visible spectrum (the vertical scales in Fig. 1 are identical), largely due to the presence of intraligand charge transfer states (*vide infra*), and an increased effective conjugation length.

The introduction of strongly donating groups in the ligand framework—leading in some cases to intraligand charge transfer excited states—provides an avenue to enhance the light absorption efficiency of transition metal complexes in general. Whilst most emphasis has been given to MLCT excited states in the literature, the design of complexes with bright ILCT transitions will improve their photophysical and photochemical properties.

### B. Orbital energies and redox potentials

**Fig. 2:**
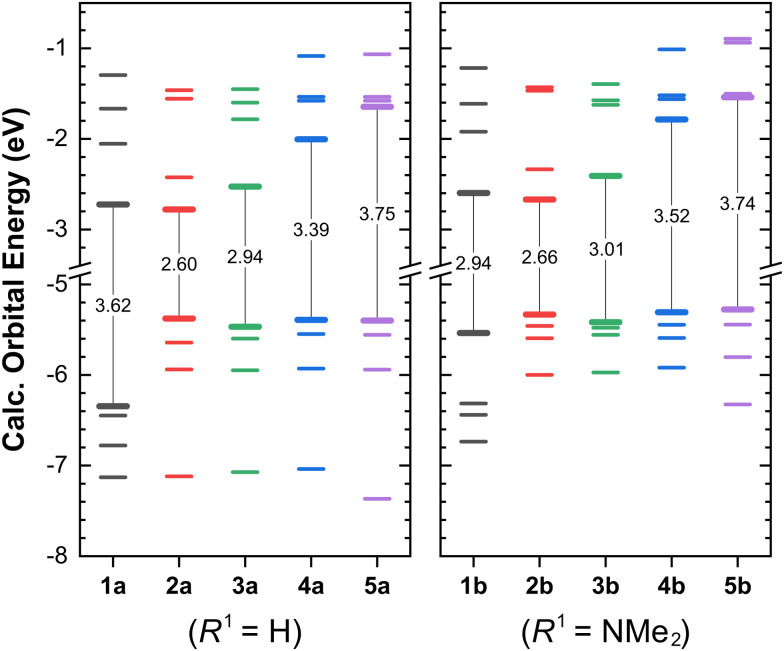
Calculated energies of the frontier orbitals of complexes (1–5)a (*R*^1^ = H, left) and (1–5)b (*R*^1^ = NMe_2_, right). The orbitals showing range from HOMO−3 to LUMO+3. Vertical lines indicate the HOMO–LUMO gap, with the corresponding values indicated in eV.

We now turn our attention to the frontier orbitals of these complexes, their localisation and their energies (Fig. 2).

On moving from 1 to 2, the energies of the LUMO levels remain largely unchanged, but the energy of the HOMO increases by *ca.* 1 eV in 2a, and only ∼0.2 eV in 2b (compared to those of 1a and 1b, respectively). We attribute this change in the HOMO to the replacement of one C≡O ligand and coordination of the additional pyridine. Fig. S12 and S13 in the ESI,† illustrate the frontier molecular orbitals of complexes 1a-b and 2a-b, respectively.

In the NMe_2_-substituted complex 1b, the HOMO is already destabilised by the electron-rich substituent in the terpyridine ligand, and localised mostly on the 4′-phenyl ring of the ligand. We observe in this case that the HOMO−1 (localised on the {Re(CO)_3_}^+^ moiety, see Fig. S12 in the ESI†) and the HOMO of 2b have a similar 1 eV difference, and that these two orbitals are almost isoenergetic with the HOMO levels of their corresponding unsubstituted counterparts (1a and 2a, respectively), showing that this effect is substituent-independent.

In the sequence 2 → 3 → 4 → 5, the HOMO levels remain largely unchanged, but the LUMO energies increase gradually with both substituents. These shifts show that ligand conjugation plays a significant role in stabilising the LUMO, and illustrate a potential structural handle to control their energies.^28^

A complementary approach to the stabilisation of ligand-centred π* LUMO levels involves the introduction of electron-withdrawing groups, as has been previously pointed out by Dempsey and co-workers.^28^ Similarly, the destabilisation of the HOMO is realised upon going from series 1 to 2 by replacing an equatorial C≡O ligand (instead of an axial C≡O ligand, as in ref. 28).

Overall, this shows that both axial and equatorial C≡O ligands provide a useful framework to tune the HOMO energies; and that both an increased conjugation and the introduction of electron withdrawing groups can stabilise the LUMO levels. With strongly electron-donating substituents introduced in a remote position of the ligand, the HOMO can be shifted from the {Re(CO)_3_}^+^ moiety to the ligand backbone, which has dramatic effects in the excited state characters and lifetimes.^24^

As we have reported previously,^24,25^ and in agreement with the results obtained from similar complexes by the groups of Dempsey^28^ and Hanan,^33^ dicarbonyl complexes like those of the 2-series are stronger photo-reductants than those of the 1-series, whilst the latter are stronger photo-oxidants. In the ground state, 1a-b and 2a-b have a similar reduction potential, but the oxidation potential of the latter is significantly lower (by at least ∼0.8 V, considering data from similar complexes in acetonitrile).^33^

To qualitatively estimate the redox potentials of complexes 3–5, we correlated the HOMO and LUMO energies of all complexes with the experimentally obtained redox potentials of similar complexes from our previous work (Fig. S14, ESI†),^24,25^ and used this to estimate the potentials of all complexes in the present work (Table 1). This approach has been previously shown to accurately reproduce experimental redox potentials in polycyclic aromatic hydrocarbons,^78^ and in Re(i) diimine carbonyl complexes.^79^

**Table 1:** Calculated redox potentials from DFT correlations

Complex	LUMO (eV)	*E* ^0^_red,calc_ (V *vs.* Fc^+/0^ in DMF)
1a	−2.72	−1.72
2a	−2.78	−1.68
3a	−2.53	−1.87
4a	−2.00	−2.25
5a	−1.65	−2.51
1b	−2.60	−1.81
2b	−2.67	−1.76
3b	−2.41	−1.95
4b	−1.79	−2.41
5b	−1.54	−2.59

From the change in the LUMO energies, we estimate the reduction potentials to become more negative in the sequence 3 → 4 → 5, whilst the corresponding oxidation potentials are expected to remain very similar (as the HOMO energies remain largely unchanged). Complexes 5a-b would then represent very potent photo-reductants, whilst absorbing at only slightly higher energies than the reference complex, 1a.

### C. Characters and energies of the excited states

**Fig. 3:**
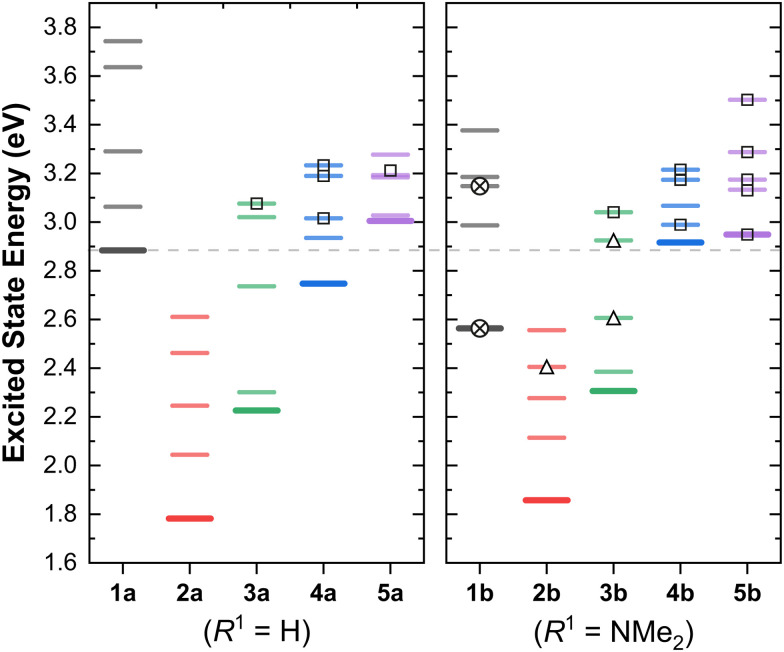
Calculated vertical transition energies of the lowest five singlet excited states of (1–5)a (*R*^1^ = H, left panel) and (1–5)b (*R*^1^ = NMe_2_, right panel), using the B3LYP functional. The horizontal dashed line shows the energy of the S_1_ state of 1a as a reference. The symbols denote the character of these transitions: MLCT (no symbol); ILCT (⊗); mixed ILCT/MLCT (Δ); MLCT to the lateral pyridine(s) (□).

Having discussed the orbital energy estimated redox potentials of these complexes, we now turn to the energies of the lowest excited states (Fig. 3).

In the two series, we observe a significant decrease of the excited state energies on going from 1 → 2. This difference, of 0.8–1 eV correlates well with the experimental red shifts (*ca.* 1.53 eV) in the lowest energy absorption bands for the MLCT complexes (*i.e.*1a*vs.*2a, and similar complexes from the Hanan group).^24,25,33^

**Fig. 4:**
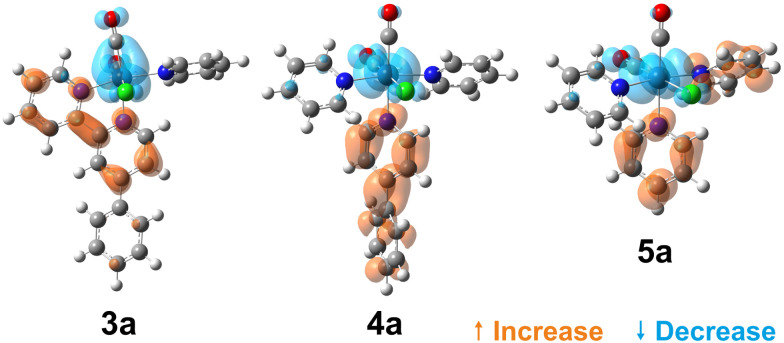
Charge density difference isosurfaces of the S_1_ ← S_0_ transition of (3–5)a, shown at |Δ*ρ*| = 0.002 a.u.

**Fig. 5:**
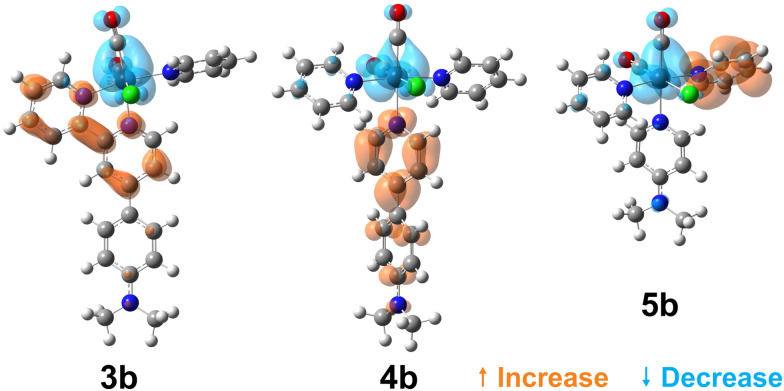
Charge density difference isosurfaces of the S_1_ ← S_0_ transition of (3–5)b, shown at |Δ*ρ*| = 0.002 a.u.

To visualise the characters of the excited states of these complexes, we now turn to the charge density difference (CDD) isosurfaces of the S_1_ ← S_0_ transition for (3–5)a (Fig. 4), and for (3–5)b (Fig. 5). Additional figures are provided in the ESI,† including detailed fragment-based excited-state character contributions and populations (Section 1.7 in the ESI†), and the CDD isosurfaces of the first 10 singlet excited states of all complexes (Section 1.8 in the ESI†).

As evidenced by the CDD isosurfaces, the lowest excited state of these complexes preserves a purely MLCT character, where the {Re(CO)_2_}^+^ moiety acts as the donor, and different combinations of the pyridine ligand orbitals act as the acceptor(s). The equatorial CO ligand (*cis* to the Cl^−^ ligand) becomes less involved in the transition as the conjugation size becomes smaller.

As evidenced in Fig. 3, we observe two ILCT transitions (to S_1_ and S_3_, respectively) in 1b, and excited states of mixed ILCT/MLCT character only for (2–3)b. In a previous report where we examined the effects of amine donor groups in organic α,β-unsaturated carbonyl dyes,^80^ we evidenced two complementary intramolecular charge transfer processes—a situation which parallels that of 1b. All other complexes show different kinds of MLCT transitions (*i.e.* to the central or lateral pyridine ligands). These results suggest that a minimum conjugation is required to access the ILCT states.

The lowest energy absorption band of MLCT character shifts from *ca.* 380 nm in 1a (*ca.* 420 nm in 1b), to *ca.* 720 nm in 2a-b. This represents a red shift of *ca.* 1.55 eV, of which ∼1 eV is accounted for by the higher energy of the HOMO orbitals in 2a-b (Fig. 2). The remaining shift is attributed to the increase in conjugation length of the system due to the planarisation of the terpyridine ligand.

Evidence in favour of this hypothesis comes from analysing the effect of the conjugation size in the energies of the excited states, and is illustrated in Fig. 3. The difference between the energies of the S_1_ states of 2a and 3a is approx. 0.44 eV, which combined with the ∼1 eV red shift attributed to the shifts in orbital energy (going from 1a to 2a) suffices to explain the experimentally observed red shift of *ca.* 1.55 eV in the absorption energies of the S_1_ state of the *κ*^3^*N*-dicarbonyl complexes (2a-b), in line with our previous results.^25^ A further blue shift of *ca.* 0.5 eV is observed in the sequence 3 → 4 for both substituents.

The energy of the lowest singlet states in the 4b → 5b sequence remain largely unchanged, whilst upon going from 4a to 5a there is an increase of *ca.* 0.25 eV with the smaller ligand. Hence, this illustrates the relative energetic contribution of each ring and the conjugation between them in the linear optical properties of these complexes.

### D. Non-linear (two-photon) absorption spectra: experiment and theory

**Fig. 6:**
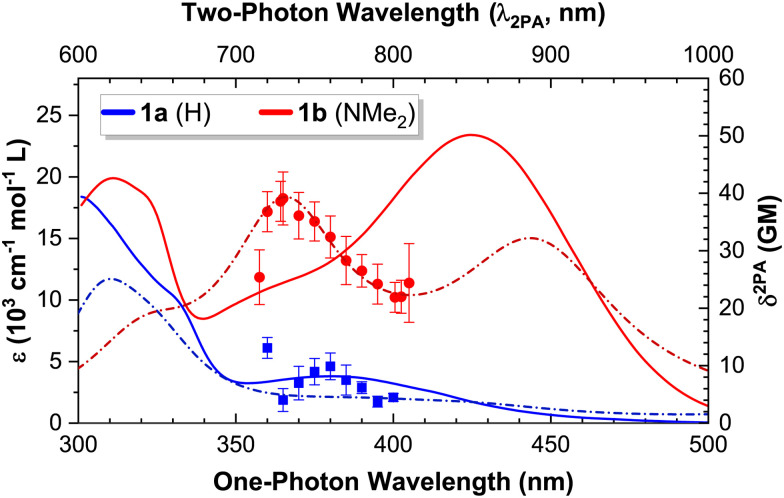
Linear (1PA) and non-linear (2PA) absorption spectra of complexes 1a and 1b: experimental 1PA extinction coefficients (solid lines; left Y scale) and 2PA cross sections (symbols = experimental data, dash-dotted lines = theoretical spectra; right Y scale). The two-photon wavelength (*λ*_2PA_ = 2 × *λ*_Abs_) corresponds to the wavelength of the 2PA experiments.

Having discussed the linear absorption properties and state energies, we now turn to the non-linear absorption properties of these complexes. We used the open aperture Z-scan technique^53^ to measure the 2PA cross section for compounds 1a and 1b in the region between 720–810 nm, where no 1PA absorption takes place (experimental data and fits are provided in the ESI,† Fig. S1–S8). The experimentally measured 2PA band corresponds in both cases to the two-photon excitation of an MLCT transition (Fig. 6).^24^ An overlay of the experimental 2PA data with the deconvolved 1PA absorption spectra is presented in the ESI† (Fig. S9 and S10).

An excellent agreement between the experimental results and the theoretical 2PA absorption spectra was obtained in both cases. The data of 1b was used to estimate the linewidth, intensity scaling and energy shifting parameters (as described in the Experimental section) that were used to generate all theoretical 2PA spectra by convolution with a Lorentzian profile. The theoretically calculated transition energies and 2PA cross sections are provided in the ESI,† Table S2.

The magnitude of the experimental 2PA cross sections at the 2PA maxima (13 GM for 1a and 39 GM for 1b) compares favourably with those reported for other d^6^ polypyridyl complexes, such as [Ru(tpy-stilbene)_2_]^2+^ (12 GM at 740 nm in MeCN) and [Ir(tpy-stilbene)_2_]^3+^ (67 GM at 740 nm in MeCN); where tpy-stilbene = 4′-(4-styrylphenyl)-2,2′:6′,2′′-terpyridine.^81^

The experimental determination of the 2PA cross sections of complexes 2a-b was greatly limited by their low solubility in DMF. Attempts to record the 2PA cross sections in DMF and in other solvents such as DCM and DMSO were also unsuccessful. The measurement of the 2PA cross section of these complexes was further complicated by the presence of low-lying absorption bands extending to *ca.* 750 nm. It is well known that in cases of 1PA absorption in the vicinity of the 2PA bands of interest, an intense pre-resonance effect can dominate over the much less efficient 2PA process, often leading to a resonance enhancement.^82,83^ In the same fashion, attempts to record the 2PA cross sections of the terpyridine ligands (La and Lb) in DMF were unsuccessful due to the generation of a stimulated Raman scattering response from the solvent at wavelengths below 700 nm under our experimental conditions.

**Fig. 7:**
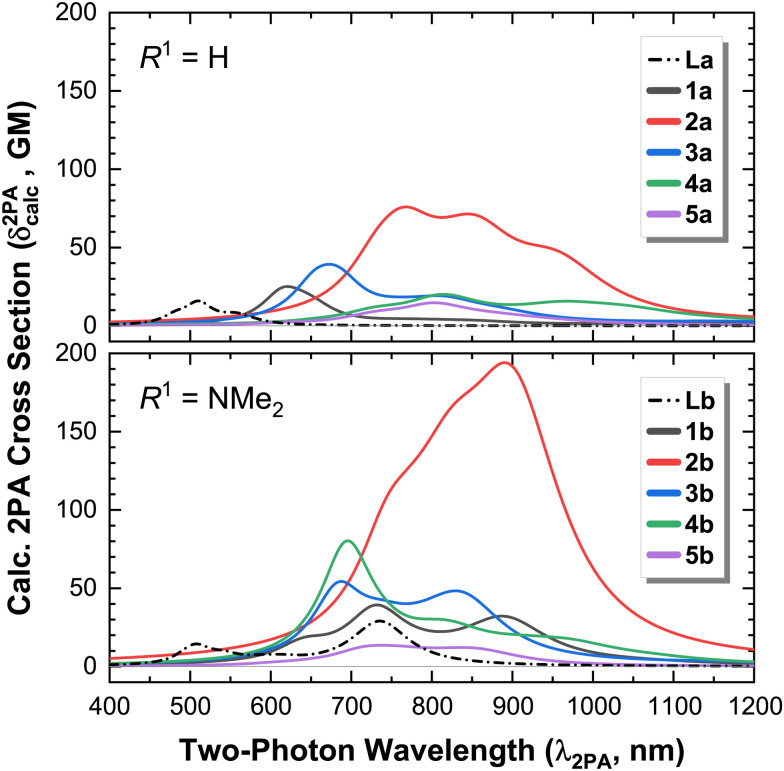
Calculated two-photon absorption (2PA) spectra of complexes (1–5)a, and ligand La (*R*^1^ = H, top); and complexes (1–5)b and ligand Lb (*R*^1^ = NMe_2_, bottom). The vertical scale of both figures is identical to facilitate comparison.

Given the excellent agreement between the 2PA experimental and calculated spectra (Fig. 6), we conclude that the chosen level of theory suffices to theoretically study the remaining complexes (2a-b; and 3–5 of both series), and focus our attention on the theoretical results. The convolved theoretical two-photon absorption spectra of all complexes are shown in Fig. 7.

To put these results in context and examine the trends and the origins of the differing 2PA cross sections, we will rely on a simplified two-state model for the maximum 2PA cross section, which has been proposed in the literature and applied to study several dye families.^84,85^ According to this model eqn (8), both the change in permanent dipole moment upon photoexcitation (Δ*μ* = *μ*_1_ − *μ*_0_) and the transition dipole moment (*M*_01_) contribute equally to the maximum 2PA cross section (*δ*^2PA^_max_), given by:8
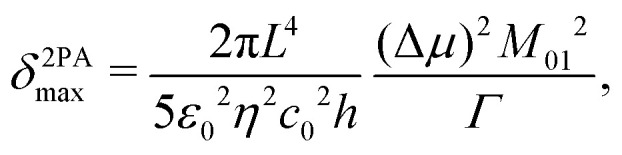
where *ε*_0_ is the vacuum permittivity, and *L* = (*η*^2^+ 2)/3 is the Lorentz local-field factor; all constants and *δ*^2PA^_max_ are in SI units.

We shall discuss the contribution of Δ*μ* and *M*_01_ separately, although the two properties are somewhat intertwined. In first place, intramolecular charge transfer (ICT) and ILCT transitions are typically associated with a larger Δ*μ* compared to π,π* or MLCT transitions (in organic and coordination compounds, respectively)—since the former typically involve greater donor–acceptor distances. Substitution of terpyridine ligands with triarylamine donor groups has been shown to increase the intramolecular charge transfer (ICT) character (leading to a larger Δ*μ*).^86^

This effect is evidenced by the slightly larger and red-shifted ICT band observed for Lb compared to the pure π,π* bands of La (Fig. 7), and is maintained upon complexation with Re(i): the 2PA cross sections of La and Lb are slightly increased in their *κ*^2^*N*-Re(i) complexes (1a-b).

We attribute this slight increase to the change from π,π* (La) to MLCT (1a), and from ICT (Lb) to ILCT (1b)—further increasing Δ*μ* and hence *δ*^2PA^_max_. These changes are also accompanied by a global red shift of the absorption bands in both cases, in agreement with the known enhancement of the electron-accepting character of the terpyridine and an extension of conjugation upon binding to a metal.^44^

The MLCT bands of complexes in the b-series are slightly shifted to higher energies with respect to those of their counterparts in the a-series (Fig. 1, 3 and 7), since the NMe_2_ substituent raises the π* orbitals of the ligand, increasing the energy gap between the metal (donor), and ligand π* (acceptor) orbitals, and hence the transition energy. Also, the strong electron donating character of the NMe_2_ group is responsible for the apparition of an additional band in complexes (1–3)b.

For the less-conjugated complex 3b, the red-shifted 2PA band corresponds to the excitation of mixed ILCT/MLCT transitions, and 4b has MLCT transitions only, as depicted in Fig. 3.

**Fig. 8:**
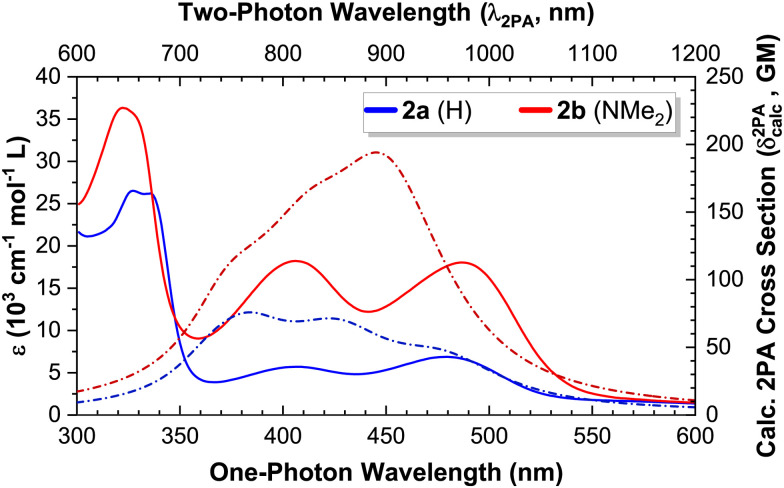
Experimental 1PA extinction coefficients (solid lines, left scale) *vs.* calculated 2PA cross sections (dash-dotted lines, right scale) for complexes 2a-b.

The effect of an increased 1PA oscillator strength—and hence of *M*_01_—is best illustrated by directly comparing complexes 2a and 2b. Whilst both possess excited states of predominantly MLCT character, the higher 1PA extinction coefficients in 2b also lead to slightly higher 2PA cross sections (which scale by roughly the same amount, Fig. 8).

**Fig. 9:**
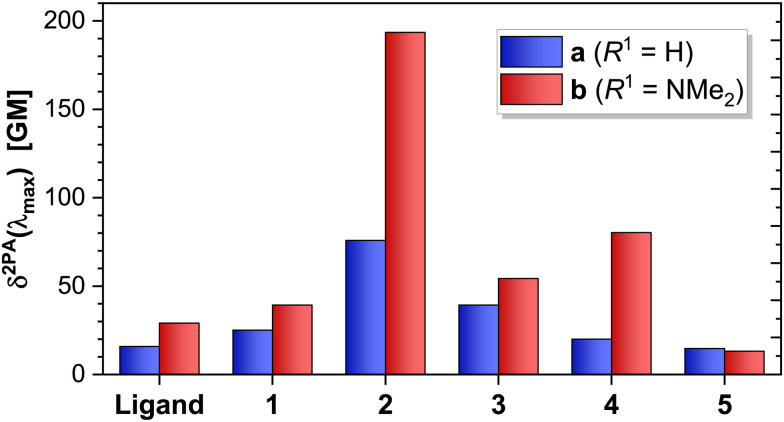
Calculated *δ*^2PA^ values at the maximum 2PA wavelength for ligands La and Lb, and all complexes studied in this work. The *δ*^2PA^_max_ values were extracted from the convoluted spectra at the maximum 2PA wavelength.

When comparing compounds 3–5 of both series, we observe that a decrease in the conjugation of the ligand further decreases the magnitude of the 2PA cross section (Fig. 9). Complex 4b constitutes a special case: albeit its excited states have a pure MLCT character (contrasting the mixed ILCT/MLCT character of some states in 2b and 3b), the lowest excited states of 4b are closer in energy as a consequence of the strongly-donating NMe_2_ group (Fig. 3). This in turn leads to an increase in the overlap of such states, whose envelopes add up and translate into a higher effective 1PA extinction coefficient (Fig. 1) and 2PA cross section (Fig. 7). This effect is absent in 4a, where the states are more well-separated instead.

Of the complexes studied herein, 2b shows the largest overall theoretical 2PA cross section, since it contains a large conjugated system due to the planarity of the *κ*^3^*N*-coordinated terpyridine, and a slightly higher ILCT character of the excited states, resulting in increased oscillator strength. These results show that a large conjugated system is more beneficial for 2PA than an I(L)CT transition: whilst the 1PA extinction coefficients for both 1b and 2b are of similar magnitude (*ca.* 2 × 10^4^ cm^−1^ mol^−1^ L), *δ*^2PA^_max_ of 2b is *ca.* 5 times larger than that of 1b; whilst the *δ*^2PA^_max_ of 2a is *ca.* 3 times larger than that of 1a.

From these results, it can be observed that there is an interplay between the MLCT and the ILCT transitions, and though the presence of an ILCT generates an additional band in the 2PA spectra, this band has roughly the same 2PA cross section associated with the MLCT band. Moreover, the 2PA cross section increases in the case of a mixed MLCT/ILCT transition (*i.e.*, 4b > 3b > 1b). Our results indicate that a higher degree of planarisation of the complex—with a consequential increase in the conjugation length—play more significant roles than the character of the transition towards increasing the magnitude of the 2PA cross-section.

Finally, as exemplified by the works of Dempsey and co-workers,^28^ and Hanan and co-workers,^33^ the axial ligand provides further potential for synthetic control of the linear and non-linear optical properties of these complexes, with promising applications towards two-photon cell imaging and photodynamic therapy.

## IV. Conclusions

In conclusion, we studied both theoretically and experimentally the linear (1PA) and non-linear (2PA) optical properties of a series of complexes based on terpyridine ligands and their derivatives.

An excellent agreement was found between the experimental and theoretical 2PA spectra of the *κ*^2^*N* tricarbonyl complexes, showing that the chosen level of theory is adequate to describe the non-linear properties of these systems.

By changing the size of the aromatic system but keeping a similar environment around the metal centre, we showed that the major contribution to enhanced 2PA cross sections comes from a large conjugated system (which allows for a larger delocalisation in the excited state). An increased charge-transfer character was shown to play a secondary role in increasing 2PA cross sections of these photoactive *d*^6^ coordination complexes.

Whilst the 1PA extinction coefficients for an ILCT transition in the *κ*^2^*N*-terpy NMe_2_-substituted complex (1b) are much higher than those of the unsubstituted complex (which has an MLCT transition), their corresponding 2PA cross sections (as predicted by theoretical calculations using singlet quadratic response) do not scale to the same extent. This complex, however, is very promising for photodynamic therapy and imaging applications since it has been previously shown to have a very long excited-state lifetime.^24^

By determining the effects of conjugation and electronic density in a remote position of the ligand framework, our results provide useful guidelines towards the design and synthesis of more efficient two-photon absorbers, predicted to be strongly photoactive complexes (with potentially long excited state lifetimes).

## Conflicts of interest

The authors declare no competing financial interest.

## Supplementary Material

CP-024-D2CP03844G-s001

CP-024-D2CP03844G-s002

## References

[cit1] Wrighton M., David L. (1974). J. Am. Chem. Soc..

[cit2] Luong J. C., Nadjo L., Wrighton M. S. (1978). J. Am. Chem. Soc..

[cit3] Luong J. C., Faltynek R. A., Wrighton M. S. (1979). J. Am. Chem. Soc..

[cit4] Hawecker J., Lehn J. M., Ziessel R. (1984). J. Chem. Soc., Chem. Commun..

[cit5] Takeda H., Koike K., Inoue H., Ishitani O. (2008). J. Am. Chem. Soc..

[cit6] Takeda H., Ishitani O. (2010). Coord. Chem. Rev..

[cit7] Kumar B., Llorente M., Froehlich J., Dang T., Sathrum A., Kubiak C. P. (2012). Annu. Rev. Phys. Chem..

[cit8] Morimoto T., Nakajima T., Sawa S., Nakanishi R., Imori D., Ishitani O. (2013). J. Am. Chem. Soc..

[cit9] Kou Y., Nabetani Y., Masui D., Shimada T., Takagi S., Tachibana H., Inoue H. (2014). J. Am. Chem. Soc..

[cit10] Schreier M., Gao P., Mayer M. T., Luo J., Moehl T., Nazeeruddin M. K., Tilley S. D., Grätzel M. (2015). Energy Environ. Sci..

[cit11] Qiu L. Q., Chen K. H., Yang Z. W., Ren F. Y., He L. N. (2021). Chem. – Eur. J..

[cit12] Yi X., Zhao J., Sun J., Guo S., Zhang H. (2013). J. Chem. Soc., Dalton Trans..

[cit13] Patrocinio A. O. T., Frin K. P., Murakami Iha N. Y. (2013). Inorg. Chem..

[cit14] Gao Y., Sun S., Han K. (2009). Spectrochim. Acta, Part A.

[cit15] El Nahhas A., Consani C., Blanco-Rodríguez A. M., Lancaster K. M., Braem O., Cannizzo A., Towrie M., Clark I. P., Záliš S., Chergui M., Vlček A. (2011). Inorg. Chem..

[cit16] Anfuso C. L., Snoeberger R. C., Ricks A. M., Liu W., Xiao D., Batista V. S., Lian T. (2011). J. Am. Chem. Soc..

[cit17] Liu J., Jiang W. (2012). Dalton Trans..

[cit18] Abdellah M., El-Zohry A. M., Antila L. J., Windle C. D., Reisner E., Hammarström L. (2017). J. Am. Chem. Soc..

[cit19] Probst B., Guttentag M., Rodenberg A., Hamm P., Alberto R. (2011). Inorg. Chem..

[cit20] Guttentag M., Rodenberg A., Kopelent R., Probst B., Buchwalder C., Brandstätter M., Hamm P., Alberto R. (2012). Eur. J. Inorg. Chem..

[cit21] Guttentag M., Rodenberg A., Bachmann C., Senn A., Hamm P., Alberto R. (2013). Dalton Trans..

[cit22] Rodenberg A., Orazietti M., Probst B., Bachmann C., Alberto R., Baldridge K. K., Hamm P. (2015). Inorg. Chem..

[cit23] Rodenberg A., Orazietti M., Mosberger M., Bachmann C., Probst B., Alberto R., Hamm P. (2016). ChemPhysChem.

[cit24] Fernández-Terán R., Sévery L. (2021). Inorg. Chem..

[cit25] Fernández-Terán R. J., Sévery L. (2021). Inorg. Chem..

[cit26] Atallah H., Taliaferro C. M., Wells K. A., Castellano F. N. (2020). Dalton Trans..

[cit27] Smithback J. L., Helms J. B., Schutte E., Woessner S. M., Sullivan B. P. (2006). Inorg. Chem..

[cit28] Kurtz D. A., Brereton K. R., Ruoff K. P., Tang H. M., Felton G. A. N., Miller A. J. M., Dempsey J. L. (2018). Inorg. Chem..

[cit29] Morimoto T., Ishitani O. (2017). Acc. Chem. Res..

[cit30] Black D. R., Hightower S. E. (2012). Inorg. Chem. Commun..

[cit31] Frenzel B. A., Schumaker J. E., Black D. R., Hightower S. E. (2013). Dalton Trans..

[cit32] Bulsink P., Al-Ghamdi A., Joshi P., Korobkov I., Woo T., Richeson D. (2016). Dalton Trans..

[cit33] Auvray T., Del Secco B., Dubreuil A., Zaccheroni N., Hanan G. S. (2021). Inorg. Chem..

[cit34] Szłapa-Kula A., Palion-Gazda J., Ledwon P., Erfurt K., Machura B. (2022). Dalton Trans..

[cit35] Szlapa-Kula A., Małecka M., Maroń A. M., Janeczek H., Siwy M., Schab-Balcerzak E., Szalkowski M., Maćkowski S., Pedzinski T., Erfurt K., Machura B. (2021). Inorg. Chem..

[cit36] Choroba K., Maroń A., Świtlicka A., Szłapa-Kula A., Siwy M., Grzelak J., Maćkowski S., Pedzinski T., Schab-Balcerzak E., Machura B. (2021). Dalton Trans..

[cit37] Choroba K., Kotowicz S., Maroń A., Świtlicka A., Szłapa-Kula A., Siwy M., Grzelak J., Sulowska K., Maćkowski S., Schab-Balcerzak E., Machura B. (2021). Dyes Pigm..

[cit38] McLay J. R. W., Sutton J. J., Shillito G. E., Larsen C. B., Huff G. S., Lucas N. T., Gordon K. C. (2021). Inorg. Chem..

[cit39] Shillito G. E., Preston D., Traber P., Steinmetzer J., McAdam C. J., Crowley J. D., Wagner P., Kupfer S., Gordon K. C. (2020). Inorg. Chem..

[cit40] Shillito G. E., Bodman S. E., Mapley J. I., Fitchett C. M., Gordon K. C. (2020). Inorg. Chem..

[cit41] Chen Y., Guan R., Zhang C., Huang J., Ji L., Chao H. (2016). Coord. Chem. Rev..

[cit42] Moritomo H., Nakagawa K., Sugihara H., Suzuki Y., Kawamata J. (2014). Chem. Lett..

[cit43] Zhang Q., Tian X., Zhou H., Wu J., Tian Y. (2017). Materials.

[cit44] Grisanti L., Sissa C., Terenziani F., Painelli A., Roberto D., Tessore F., Ugo R., Quici S., Fortunati I., Garbin E., Ferrante C., Bozio R. (2009). Phys. Chem. Chem. Phys..

[cit45] Lakowicz J. R., Castellano F. N., Gryczynski I., Gryczynski Z., Dattelbaum J. D. (1999). J. Photochem. Photobiol., A.

[cit46] Heinemann F., Karges J., Gasser G. (2017). Acc. Chem. Res..

[cit47] McKenzie L. K., Sazanovich I. V., Baggaley E., Bonneau M., Guerchais V., Williams J. A., Weinstein J. A., Bryant H. E. (2017). Chem. – Eur. J..

[cit48] McKenzie L. K., Bryant H. E., Weinstein J. A. (2019). Coord. Chem. Rev..

[cit49] Phillips D. (1995). Pure Appl. Chem..

[cit50] Bhawalkar J. D., Kumar N. D., Zhao C. F., Prasad P. N. (1997). J. Clin. Laser Med. Surg..

[cit51] Bolze F., Jenni S., Sour A., Heitz V. (2017). Chem. Commun..

[cit52] Karges J., Blacque O., Jakubaszek M., Goud B., Goldner P., Gasser G. (2019). J. Inorg. Biochem..

[cit53] Sheik-Bahae M., Said A., Wei T.-H., Hagan D., Van Stryland E. (1990). IEEE J. Quantum Electron..

[cit54] Ajami A., Husinsky W., Liska R., Pucher N. (2010). J. Opt. Soc. Am. B.

[cit55] Frisch M. J. , Trucks G. W., Schlegel H. B., Scuseria G. E., Robb M. A., Cheeseman J. R., Scalmani G., Barone V., Petersson G. A., Nakatsuji H., Li X., Caricato M., Marenich A. V., Bloino J., Janesko B. G., Gomperts R., Mennucci B., Hratchian H. P., Ortiz J. V., Izmaylov A. F., Sonnenberg J. L., Williams-Young D., Ding F., Lipparini F., Egidi F., Goings J., Peng B., Petrone A., Henderson T., Ranasinghe D., Zakrzewski V. G., Gao J., Rega N., Zheng G., Liang W., Hada M., Ehara M., Toyota K., Fukuda R., Hasegawa J., Ishida M., Nakajima T., Honda Y., Kitao O., Nakai H., Vreven T., Throssell K., Montgomery Jr. J. A., Peralta J. E., Ogliaro F., Bearpark M. J., Heyd J. J., Brothers E. N., Kudin K. N., Staroverov V. N., Keith T. A., Kobayashi R., Normand J., Raghavachari K., Rendell A. P., Burant J. C., Iyengar S. S., Tomasi J., Cossi M., Millam J. M., Klene M., Adamo C., Cammi R., Ochterski J. W., Martin R. L., Morokuma K., Farkas O., Foresman J. B. and Fox D. J., Gaussian 16, Revision B.01, 2017, http://gaussian.com/

[cit56] Klemens T., Świtlicka A., Szlapa-Kula A., Krompiec S., Lodowski P., Chrobok A., Godlewska M., Kotowicz S., Siwy M., Bednarczyk K., Libera M., Maćkowski S., Pe dziński T., Schab-Balcerzak E., Machura B. (2018). Appl. Organomet. Chem..

[cit57] Hay P. J., Wadt W. R. (1985). J. Chem. Phys..

[cit58] Wadt W. R., Hay P. J. (1985). J. Chem. Phys..

[cit59] Hay P. J., Wadt W. R. (1985). J. Chem. Phys..

[cit60] Lee C., Yang W., Parr R. G. (1988). Phys. Rev. B: Condens. Matter Mater. Phys..

[cit61] Becke A. D. (1988). Phys. Rev. A: At., Mol., Opt. Phys..

[cit62] Becke A. D. (1993). J. Chem. Phys..

[cit63] Scalmani G., Frisch M. J. (2010). J. Chem. Phys..

[cit64] Plasser F. (2020). J. Chem. Phys..

[cit65] Plasser F., Wormit M., Dreuw A. (2014). J. Chem. Phys..

[cit66] Plasser F., Lischka H. (2012). J. Chem. Theory Comput..

[cit67] O'Boyle N. M., Tenderholt A. L., Langner K. M. (2008). J. Comput. Chem..

[cit68] Lu T., Chen F. (2012). J. Comput. Chem..

[cit69] Dalton, a molecular electronic structure program, 2020, https://daltonprogram.org/

[cit70] Aidas K., Angeli C., Bak K. L., Bakken V., Bast R., Boman L., Christiansen O., Cimiraglia R., Coriani S., Dahle P., Dalskov E. K., Ekström U., Enevoldsen T., Eriksen J. J., Ettenhuber P., Fernández B., Ferrighi L., Fliegl H., Frediani L., Hald K., Halkier A., Hättig C., Heiberg H., Helgaker T., Hennum A. C., Hettema H., Hjertenæs E., Høst S., Høyvik I. M., Iozzi M. F., Jansík B., Jensen H. J. A., Jonsson D., Jørgensen P., Kauczor J., Kirpekar S., Kjærgaard T., Klopper W., Knecht S., Kobayashi R., Koch H., Kongsted J., Krapp A., Kristensen K., Ligabue A., Lutnæs O. B., Melo J. I., Mikkelsen K. V., Myhre R. H., Neiss C., Nielsen C. B., Norman P., Olsen J., Olsen J. M. H., Osted A., Packer M. J., Pawlowski F., Pedersen T. B., Provasi P. F., Reine S., Rinkevicius Z., Ruden T. A., Ruud K., Rybkin V. V., Sałek P., Samson C. C., de Merás A. S., Saue T., Sauer S. P., Schimmelpfennig B., Sneskov K., Steindal A. H., Sylvester-Hvid K. O., Taylor P. R., Teale A. M., Tellgren E. I., Tew D. P., Thorvaldsen A. J., Thøgersen L., Vahtras O., Watson M. A., Wilson D. J., Ziolkowski M., Ågren H. (2014). Wiley Interdiscip. Rev.: Comput. Mol. Sci..

[cit71] Sałek P., Vahtras O., Helgaker T., Ågren H. (2002). J. Chem. Phys..

[cit72] Hettema H., Jensen H. J. A., Jørgensen P., Olsen J. (1992). J. Chem. Phys..

[cit73] Bouvier R., Durand R., Favereau L., Srebro-Hooper M., Dorcet V., Roisnel T., Vanthuyne N., Vesga Y., Donnelly J., Hernandez F., Autschbach J., Trolez Y., Crassous J. (2018). Chem. – Eur. J..

[cit74] Dipold J., Romero E. E., Donnelly J., Calheiro T. P., Bonacorso H. G., Iglesias B. A., Siqueira J. P., Hernandez F. E., De Boni L., Mendonca C. R. (2019). Phys. Chem. Chem. Phys..

[cit75] Silva D. L., Krawczyk P., Bartkowiak W., Mendonça C. R. (2009). J. Chem. Phys..

[cit76] Ohta K., Antonov L., Yamada S., Kamada K. (2007). J. Chem. Phys..

[cit77] Beerepoot M. T., Friese D. H., List N. H., Kongsted J., Ruud K. (2015). Phys. Chem. Chem. Phys..

[cit78] Méndez-Hernández D. D., Tarakeshwar P., Gust D., Moore T. A., Moore A. L., Mujica V. (2013). J. Mol. Model..

[cit79] Sanhueza L., Barrera M., Crivelli I. (2013). Polyhedron.

[cit80] Sucre-Rosales E., Fernández-Terán R., Urdaneta N., Hernández F. E., Echevarria L. (2020). Chem. Phys..

[cit81] Natrajan L. S., Toulmin A., Chew A., Magennis S. W. (2010). Dalton Trans..

[cit82] Meyer R. K., Shkunov M. N., Benner R. E., Gellermann W., Vardeny Z. V., Lin J., Barton T. J. (1997). Opt. Probes Conjug. Polym..

[cit83] Drobizhev M., Makarov N. S., Hughes T., Rebane A. (2007). J. Phys. Chem. B.

[cit84] Terenziani F., Katan C., Badaeva E., Tretiak S., Blanchard-Desce M. (2008). Adv. Mater..

[cit85] Zhou F. X., Zheng Z., Zhou H. P., Ke W. Z., Wang J. Q., Yu Z. P., Jin F., Yang J. X., Wu J. Y., Tian Y. P. (2012). CrystEngComm.

[cit86] Liu J., Zhu Y., Tian X., Li F., Xu W., Zhang Y., Wang C., Zhang J., Zhou H., Wu J., Tian Y. (2016). Dyes Pigm..

