# Supplementary material for: Dissecting conjugation and electronic effects on the linear and non-linear optical properties of rhenium(i) carbonyl complexes
Source: Phys Chem Chem Phys. 2022 Oct 31;24(45):28069–79. doi: 10.1039/d2cp03844g (PMC9682488; doi:10.1039/d2cp03844g)
Supplement: CP-024-D2CP03844G-s002 [file CP-024-D2CP03844G-s002.pdf]

Electronic Supplementary Material (ESI) for Physical Chemistry Chemical Physics.
This journal is © the Owner Societies 2022

SUPPORTING INFORMATION FOR:

Dissecting Conjugation and Electronic Effects in the Linear and Non-Linear Optical Properties of Rhenium(I) Carbonyl Complexes

Ricardo J. Fernández-Terán,[ab]* Estefanía Sucre-Rosales,[c] Lorenzo Echevarria,[cd] and Florencio E. Hernández[ce]

[a] *Department of Chemistry, University of Sheffield. Sheffield S3 7HF, United Kingdom.*
[b] *Department of Chemistry, University of Zurich. Winterthurerstrasse 190, CH-8057 Zurich, Switzerland.*
[c] *Department of Chemistry, University of Central Florida, Orlando, Florida 32816, United States.*
[d] *Departamento de Química, Universidad Simón Bolívar, Caracas 1080-A, AP 89000, Venezuela.*
[e] *CREOL/The College of Optics and Photonics, University of Central Florida, Orlando, Florida 32816, United States.*

**E-mail:* Ricardo.Fernandez@sheffield.ac.uk and Ricardo.FernandezTeran@gmail.com

Contents

Page

1. Additional Figures . . . S2
   1.1. Z-Scan Data for Complex **1a** in DMF . . . S2
   1.2. Z-Scan Data for Complex **1b** in DMF . . . S3
   1.3. Deconvolution of the 1PA Spectra and Experimental 2PA Data . . . S5
   1.4. Comparison of Experimental and Theoretical Absorption Spectra . . . S6
   1.5. Frontier Molecular Orbitals . . . S6
   1.6. Estimated Redox Potentials from MO Energies . . . S7
   1.7. Excited State Characters: Fragment-Based Analysis . . . S8
   1.8. Charge Density Difference Isosurfaces . . . S14
2. References . . . S24

1. Additional Figures

1.1 Z-Scan Data for Complex 1a in DMF

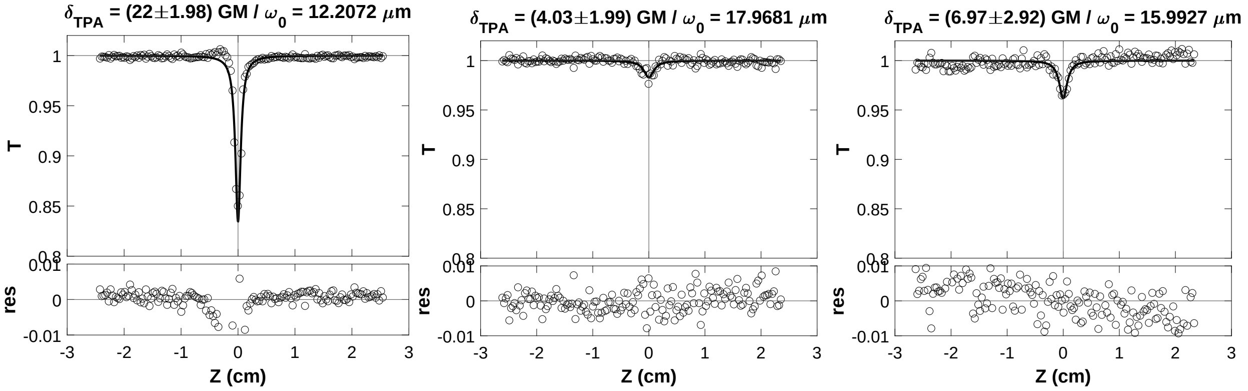

Figure S1: Z-scan data with fit and residuals for complex **1a** in DMF (5 mᴍ). Excitation wavelengths: 720 (left), 730 (centre) and 740 nm (right).

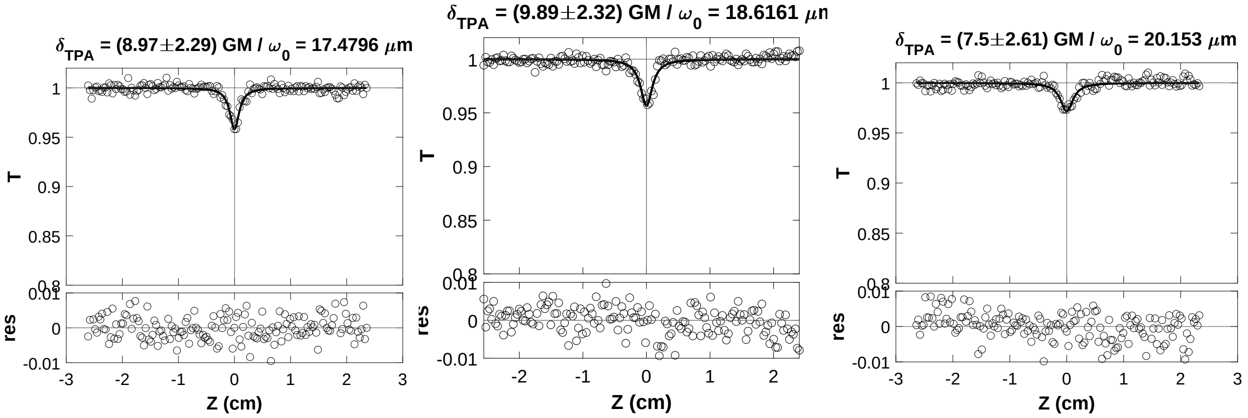

Figure S2: Z-scan data with fit and residuals for complex **1a** in DMF (5 mᴍ). Excitation wavelengths: 750 (left), 760 (centre) and 770 nm (right).

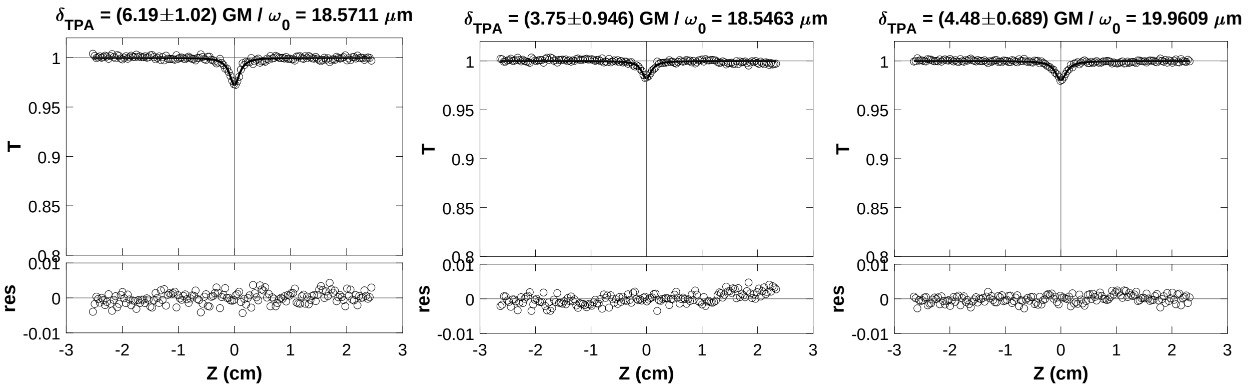

Figure S3: Z-scan data with fit and residuals for complex **1a** in DMF (5 mᴍ). Excitation wavelengths: 780 (left), 790 (centre), and 800 nm (right).

1.2 Z-Scan Data for Complex 1b in DMF

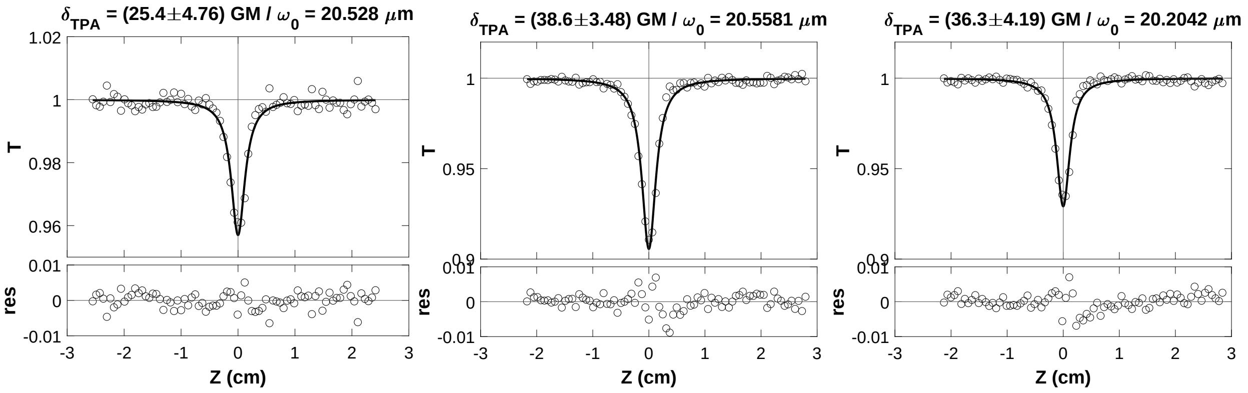

Figure S4: Z-scan data with fit and residuals for complex **1b** in DMF (5 mᴍ). Excitation wavelengths: 715 (left), 720 (centre) and 728 nm (right).

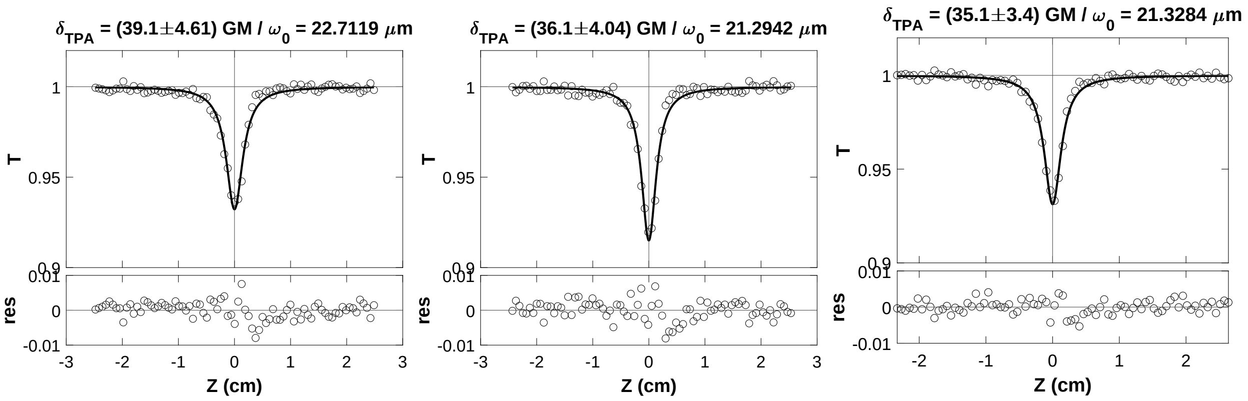

Figure S5: Z-scan data with fit and residuals for complex **1b** in DMF (5 mᴍ). Excitation wavelengths: 730 (left), 740 (centre) and 750 nm (right).

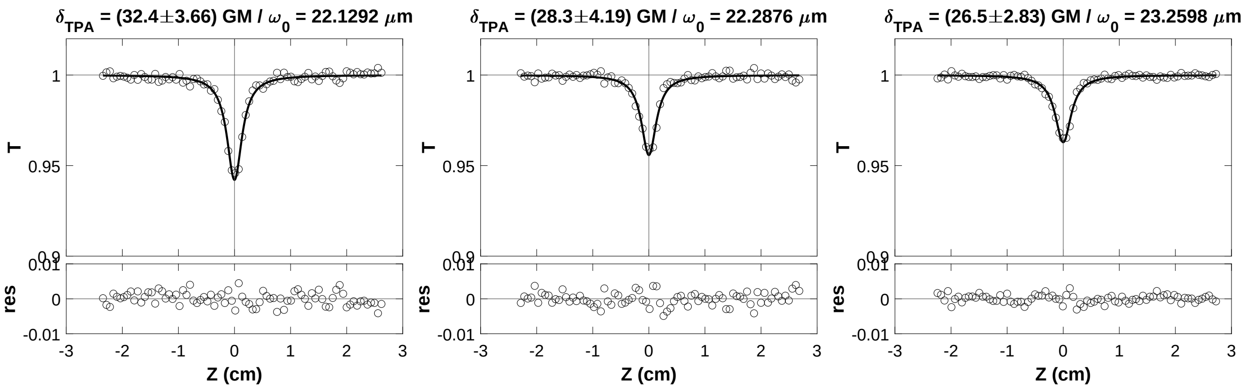

Figure S6: Z-scan data with fit and residuals for complex **1b** in DMF (5 mᴍ). Excitation wavelengths: 760 (left), 770 (centre), and 780 nm (right).

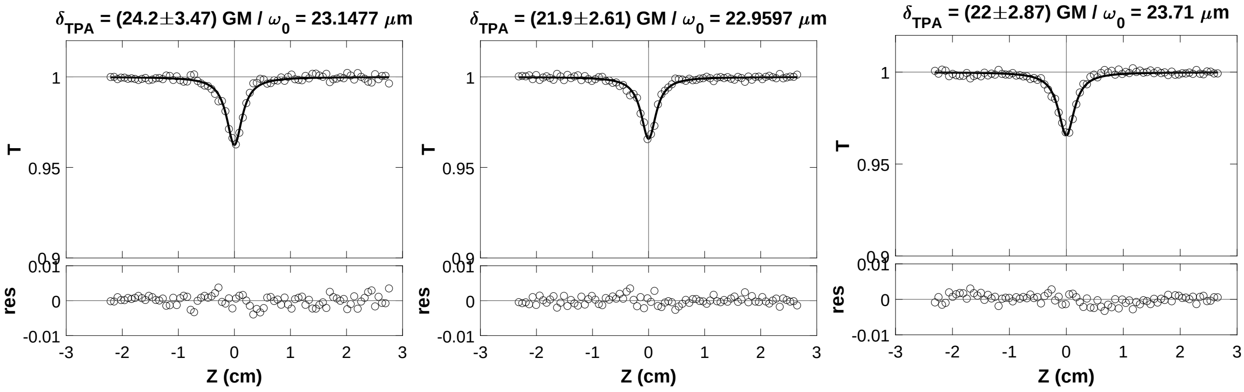

Figure S7: Z-scan data with fit and residuals for complex **1b** in DMF (5 mм). Excitation wavelengths: 790 (left), 801 (centre), and 805 nm (right).

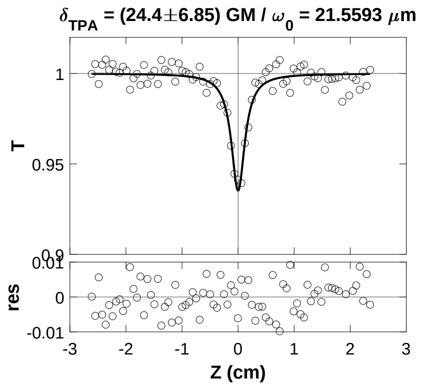

Figure S8: Z-scan data with fit and residuals for complex **1b** in DMF (5 mм). Excitation wavelength: 810 nm.

1.3 Deconvolution of the 1PA Spectra and Experimental 2PA Data

The following figures illustrate the results of the deconvolution of the 1PA absorption spectra with Gaussian functions (fitted in energy units, and plotted in wavelength), together with the experimental 2PA data. This serves to show which band(s) are being excited in the 2PA spectra, and are discussed in more depth in the manuscript. A total of four Gaussian functions was used, yielding an excellent fit to the data.

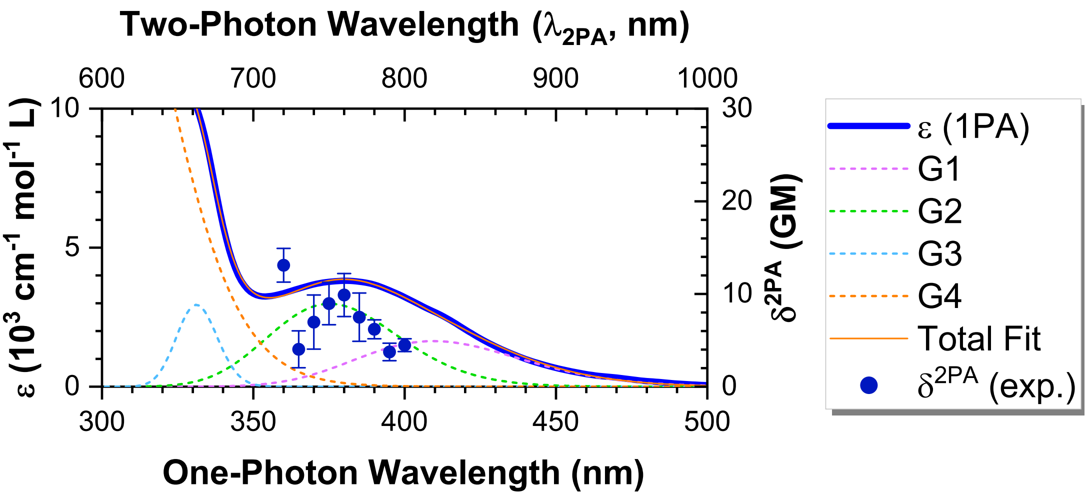

Figure S9: Deconvolution of the experimental 1PA spectra (solid lines, left scale) with Gaussian functions (dashed lines), and the experimental 2PA data (right scale) for complex **1a** in DMF. The vertical scale of the left axis has been scaled to better visualise the weaker MLCT absorption bands.

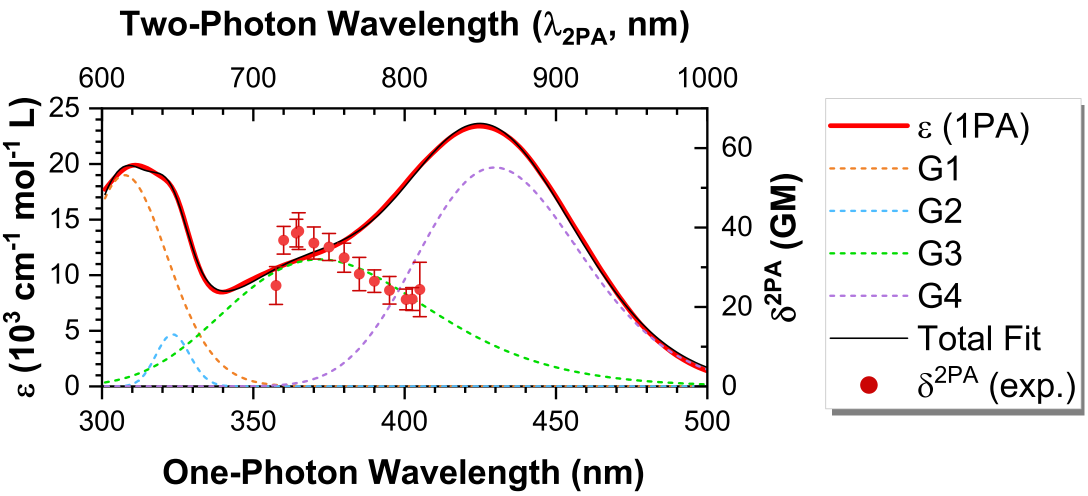

Figure S10: Deconvolution of the experimental 1PA spectra (solid lines, left scale) with Gaussian functions (dashed lines), and the experimental 2PA data (right scale) for complex **1b** in DMF.

1.4 Comparison of Experimental and Theoretical Absorption Spectra

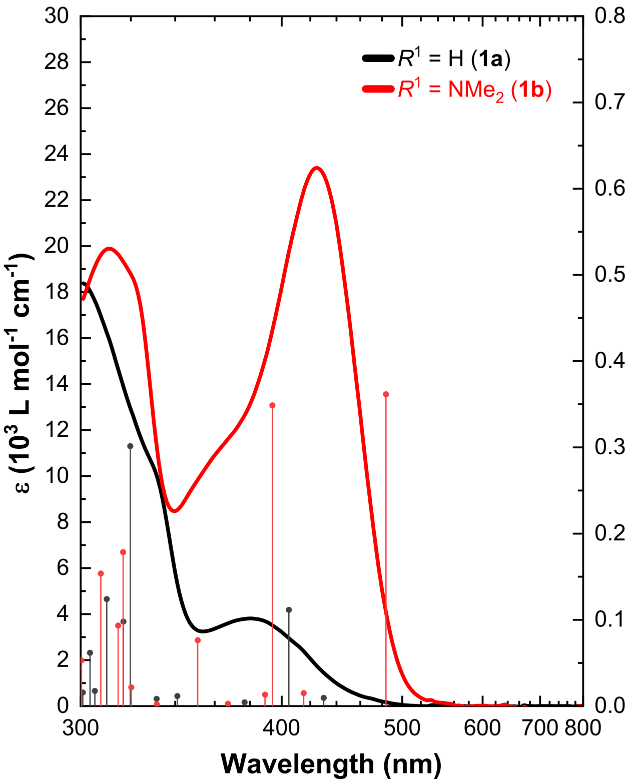

(a) Complexes **1a-b**.

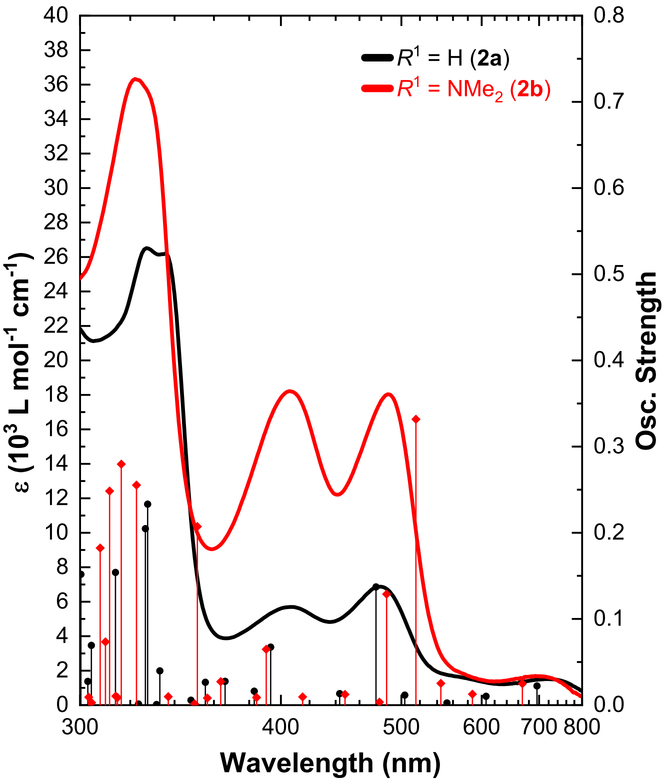

(b) Complexes **2a-b**.

Figure S11: Experimental UV-Vis absorption spectra (in DMF, solid lines) and calculated transitions and oscillator strengths.

1.5 Frontier Molecular Orbitals

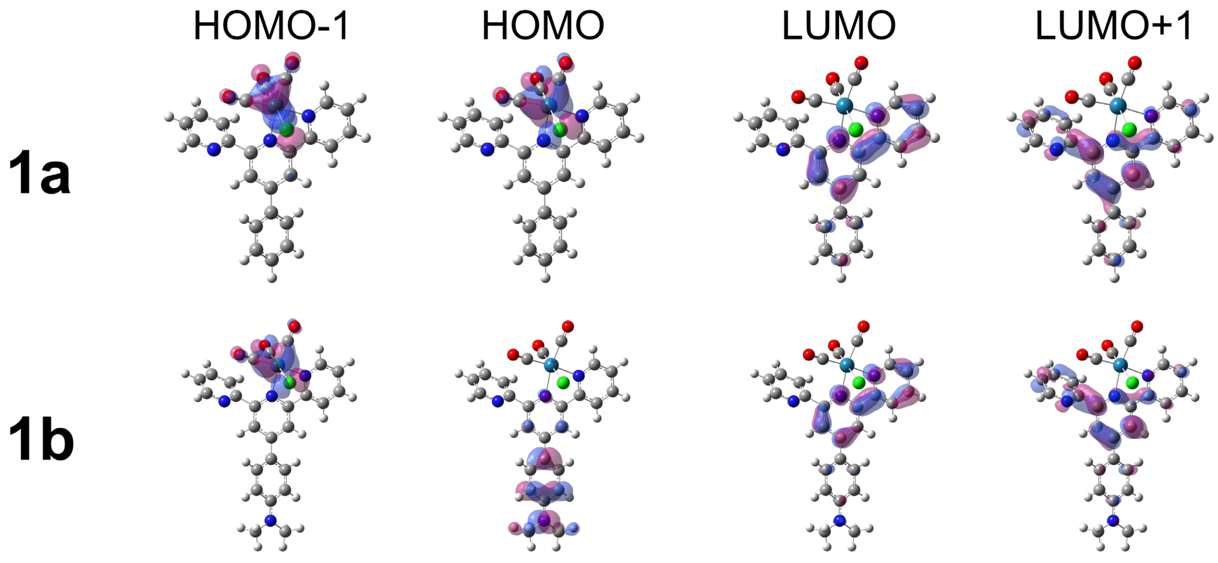

Figure S12: Frontier molecular orbitals of complexes **1a-b**, plotted at $|\rho| = 0.04$ a.u.

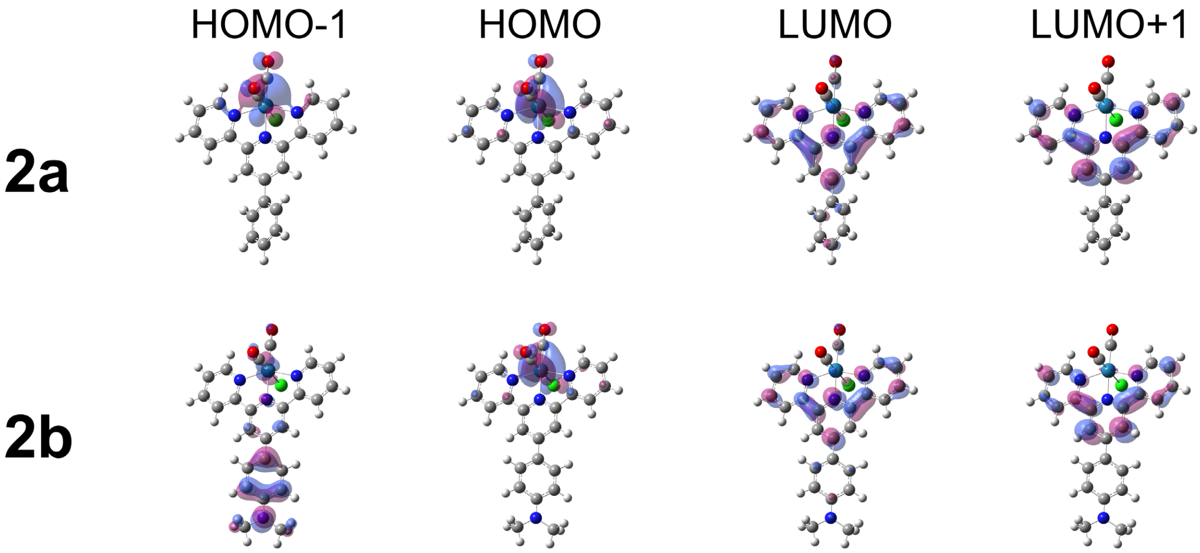

Figure S13: Frontier molecular orbitals of complexes **2a-b**, plotted at $|\rho| = 0.04$ a.u.

1.6 Estimated Redox Potentials from MO Energies

The reduction potentials of the complexes discussed in this work were calculated based on the previously reported[1] redox potentials of a series of substituted [Re(κ^2N-tpy)(CO)$_3$Cl] complexes [tpy = 4'-(4-R^1-phenyl)-2,2':6',2"-terpyridine]. We have used in the present work the same DFT functional, basis set and solvation model, thus enabling a comparison between these values and those estimated for the new complexes ().

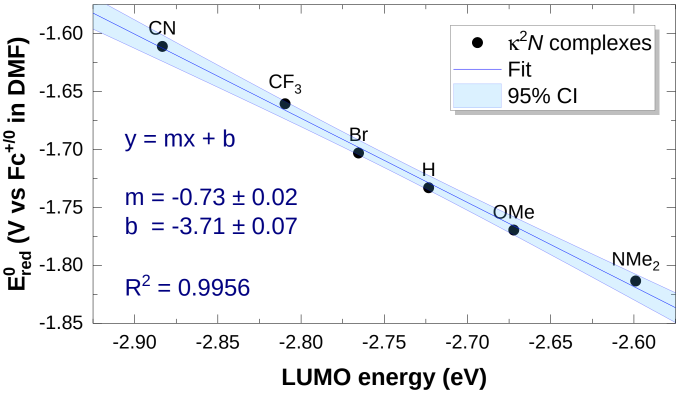

Figure S14: Correlation of the LUMO energies and experimental reduction potentials for a series of substituted [Re(κ^2N-tpy)(CO)$_3$Cl] complexes. Linear fit, extracted parameters and 95% confidence intervals shown.

1.7 Excited State Characters: Fragment-Based Analysis

To provide an in-depth perspective on the character contributions of the excited states of all complexes, we performed a fragment-based decomposition of the contributions to each excited state. The fragments are defined in Figure **S15**:

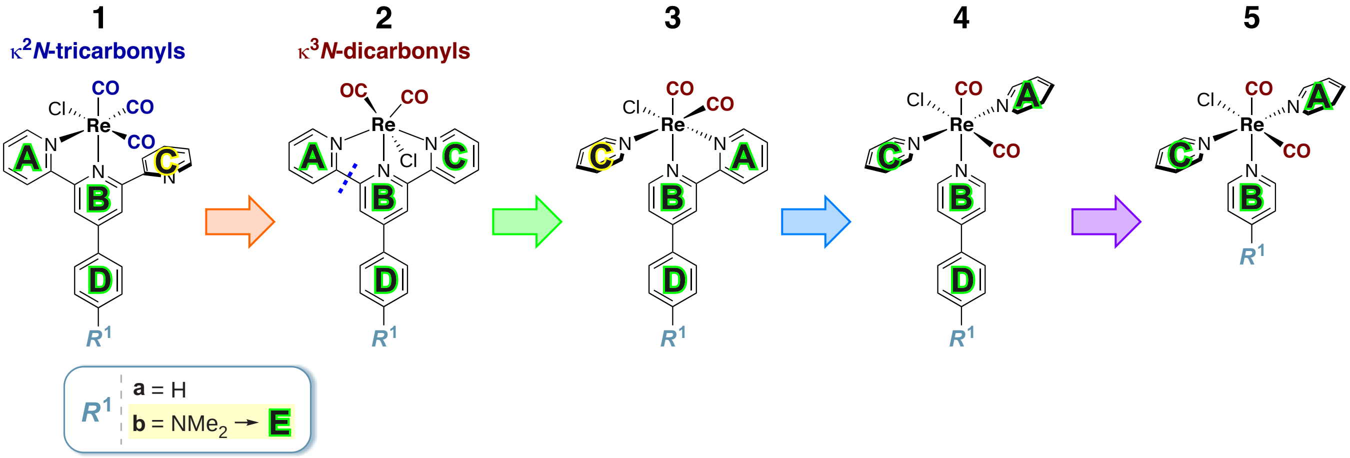

Figure S15: Fragment definition for the complexes examined in this work.

The character contributions were divided in the following 'textbook' categories, denoted $\alpha \rightarrow \beta$ (i.e. electronic density shifts from α to β):

- **MLCT** (Metal-to-Ligand Charge Transfer): Re atom $\rightarrow$ all ligand fragments.
- **ILCT** (Intra-Ligand Charge Transfer): 'Tail' of the ligand [i.e. fragments D and E (when present)] $\rightarrow$ adjacent/directly connected ligand fragments (e.g. fragments A and B in **3a**).
- **XLCT** (Halogen-to-Ligand Charge Transfer): Halogen atom $\rightarrow$ all other ligand fragments (excluding the metal).
- **LMCT** (Ligand-to-Metal Charge Transfer): All ligand fragments $\rightarrow$ Re atom.
- **CO-to-ligand**: All CO ligands $\rightarrow$ all other ligands (excluding the metal).
- **MC** (Metal-Centred): Re atom $\rightarrow$ Re atom.
- Other special cases are described on a case-specific basis in the plot and/or plot caption.

The sum of all contributions need not equal 1, since some contributions are not always considered (e.g. ligand-to-ligand charge transfers, etc.). In addition, a fragment-based electron/hole correlation plot is provided for each complex. In all cases, the corresponding fragments included all associated hydrogen atoms.

These calculations were performed in the TheoDORE program (v3.0),[2] using the inbuilt transition density matrix analysis modules.[3,4] The TheoDORE program internally uses the CCLIB library (v1.7.2)[5] to parse the output of the Gaussian calculations.

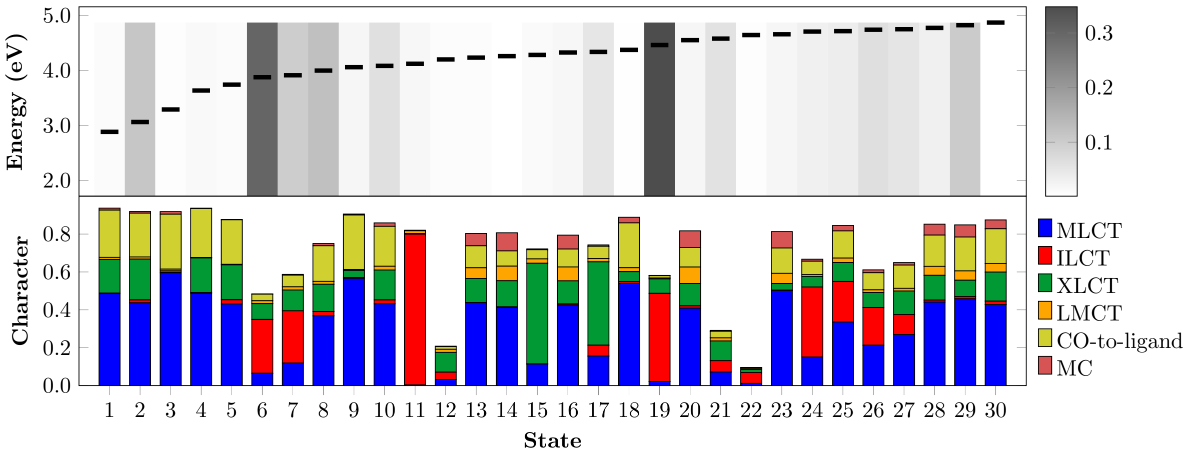

(a) Character decomposition [bottom row]; energies (horizontal lines) and oscillator strengths (as shading intensity) [top row].

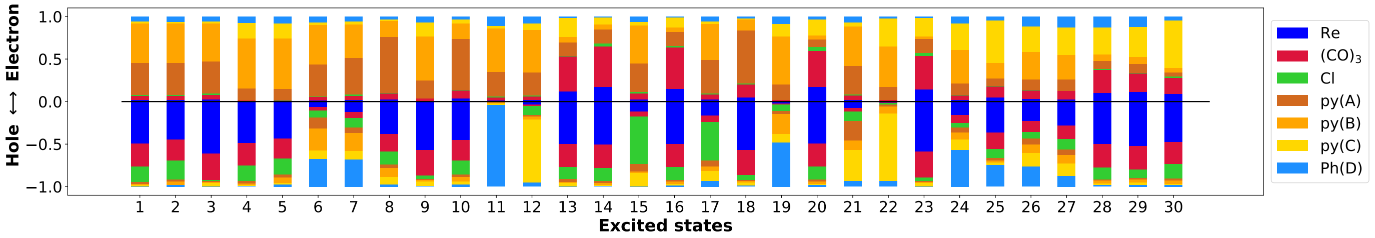

(b) Fragment decomposition of the hole/electron populations for each excited state.

Figure S16: Excited State Analysis of Complex **1a**

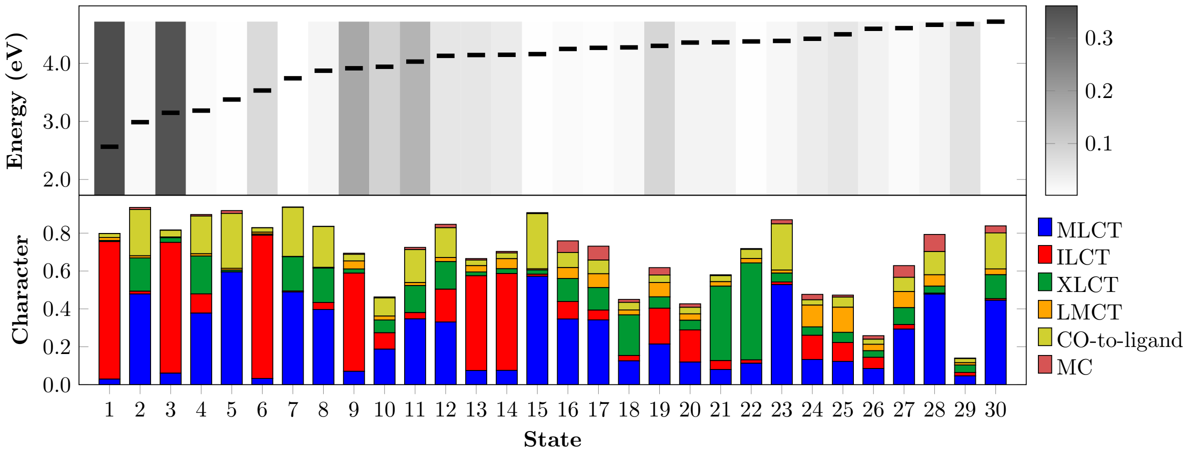

(a) Character decomposition [bottom row]; energies (horizontal lines) and oscillator strengths (as shading intensity) [top row].

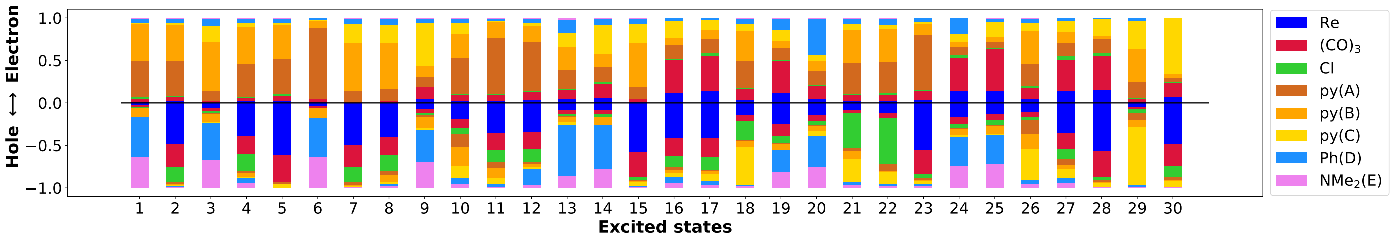

(b) Fragment decomposition of the hole/electron populations for each excited state.

Figure S17: Excited State Analysis of Complex **1b**

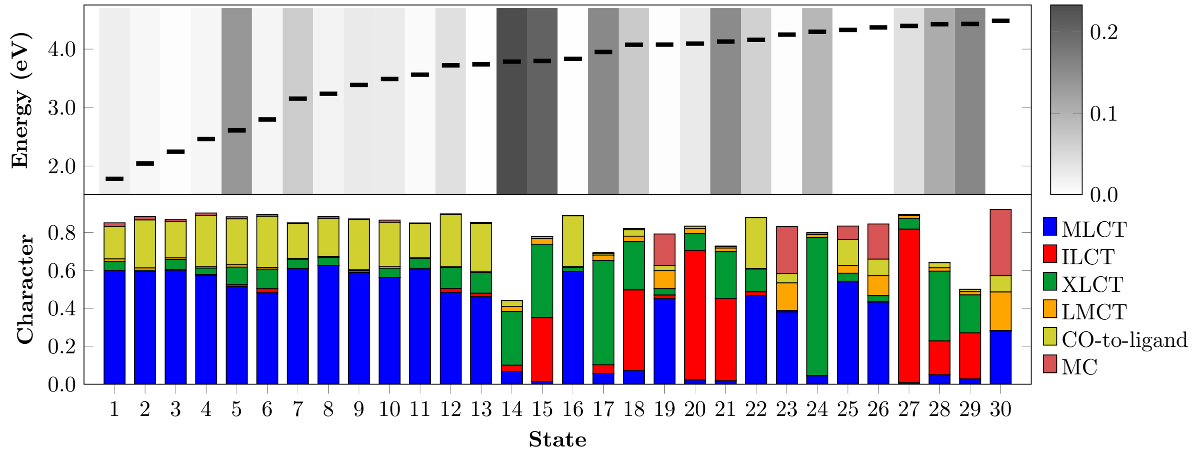

(a) Character decomposition [bottom row]; energies (horizontal lines) and oscillator strengths (as shading intensity) [top row].

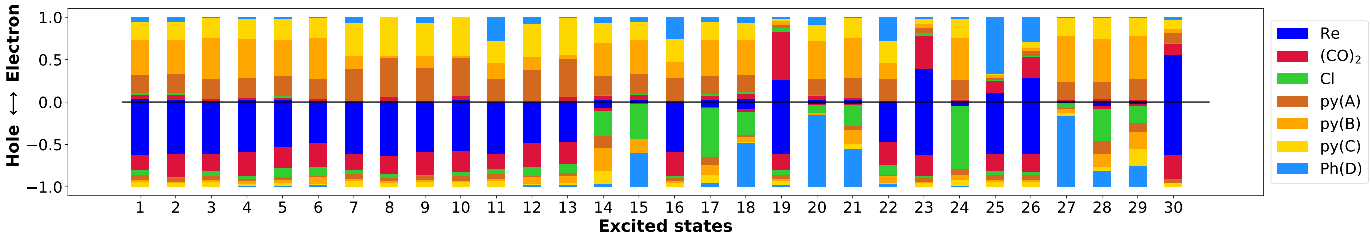

(b) Fragment decomposition of the hole/electron populations for each excited state.

Figure S18: Excited State Analysis of Complex **2a**

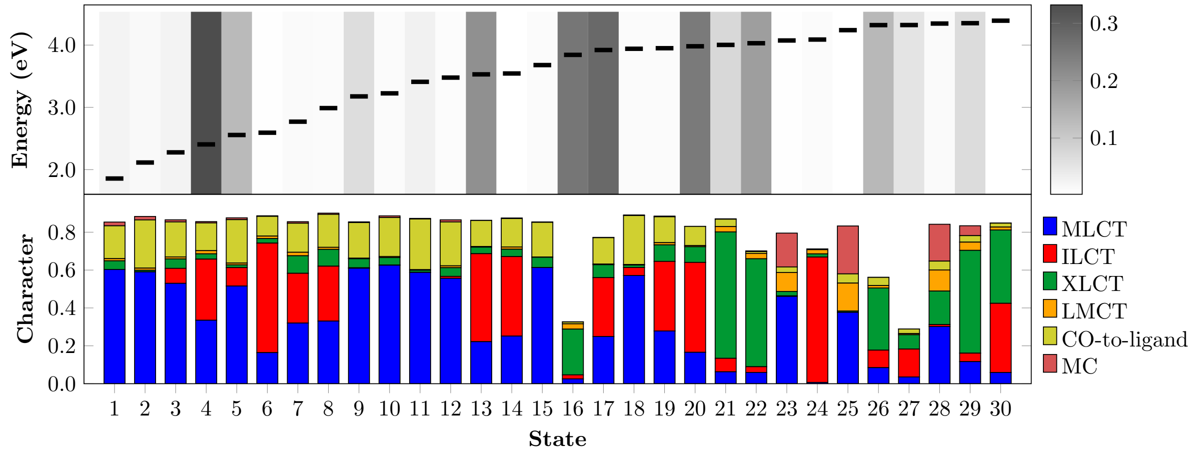

(a) Character decomposition [bottom row]; energies (horizontal lines) and oscillator strengths (as shading intensity) [top row].

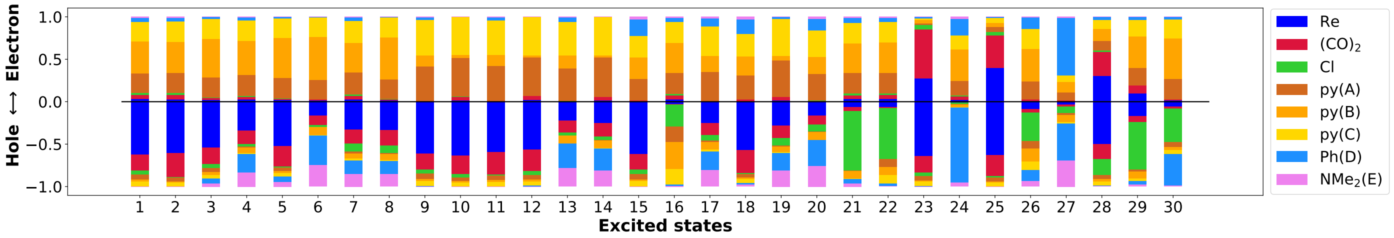

(b) Fragment decomposition of the hole/electron populations for each excited state.

Figure S19: Excited State Analysis of Complex **2b**

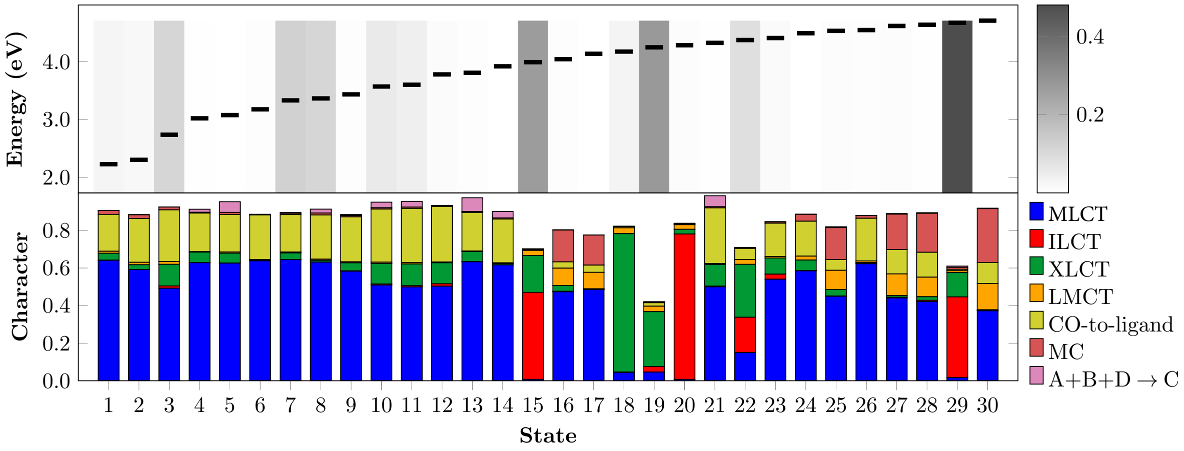

(a) Character decomposition [bottom row]; energies (horizontal lines) and oscillator strengths (as shading intensity) [top row].

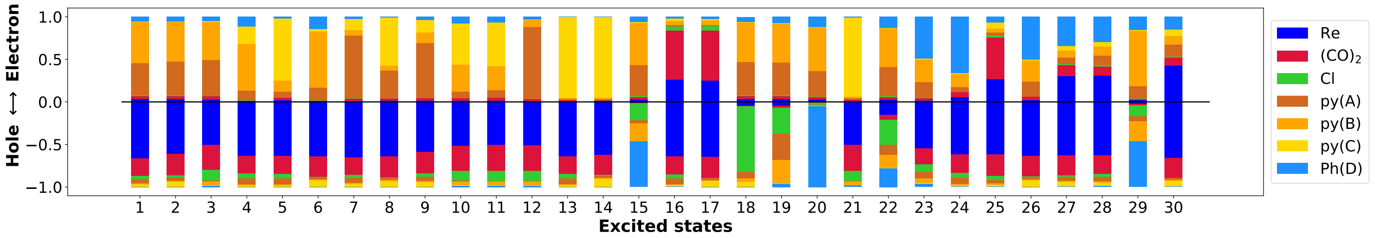

(b) Fragment decomposition of the hole/electron populations for each excited state.

Figure S20: Excited State Analysis of Complex **3a**

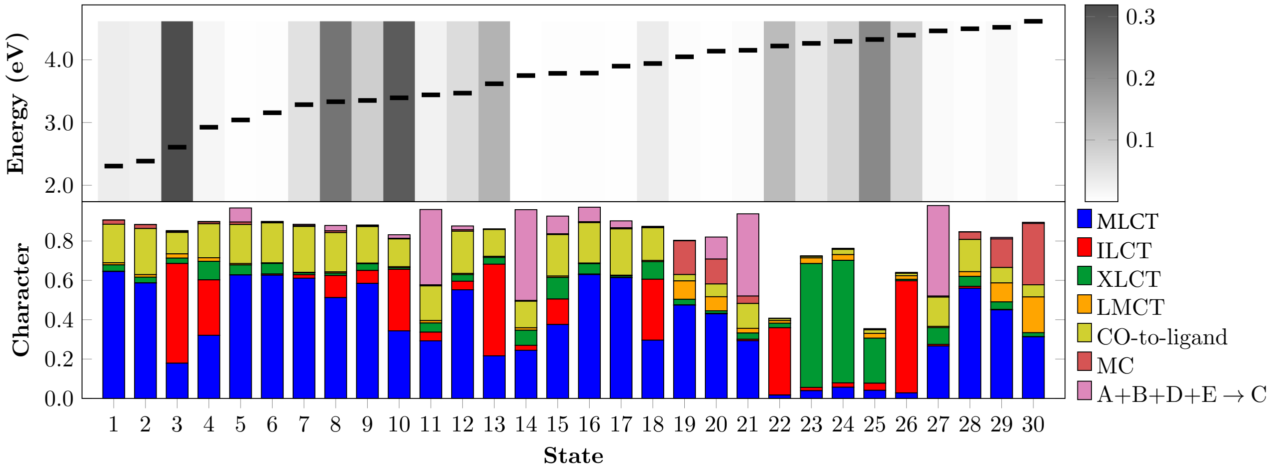

(a) Character decomposition [bottom row]; energies (horizontal lines) and oscillator strengths (as shading intensity) [top row].

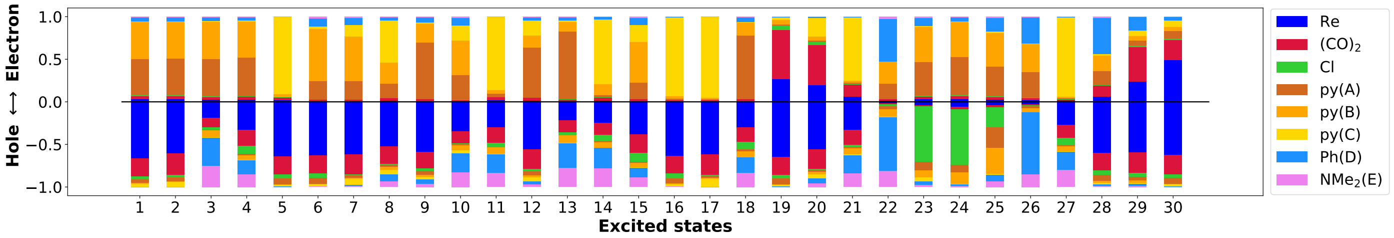

(b) Fragment decomposition of the hole/electron populations for each excited state.

Figure S21: Excited State Analysis of Complex **3b**

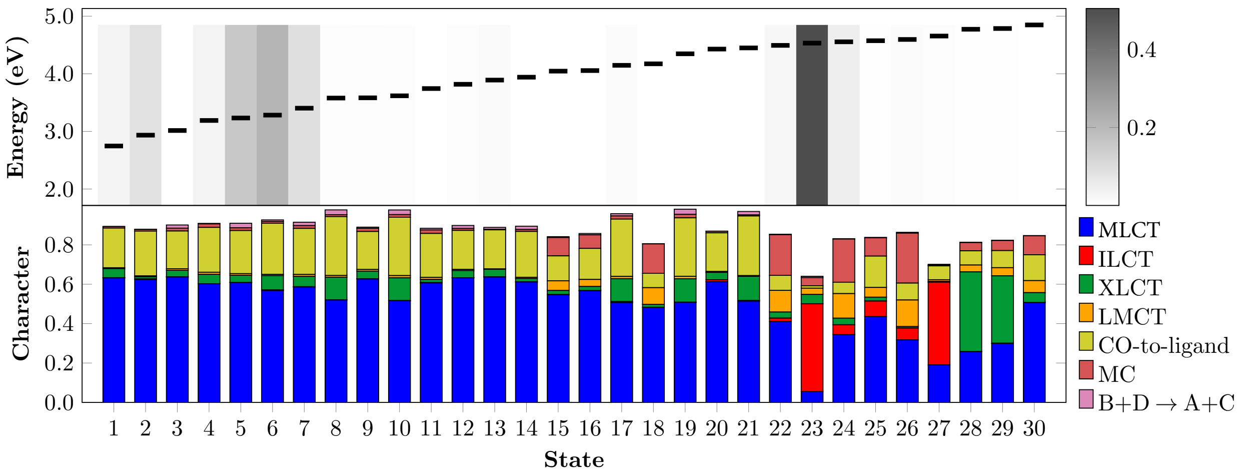

(a) Character decomposition [bottom row]; energies (horizontal lines) and oscillator strengths (as shading intensity) [top row].

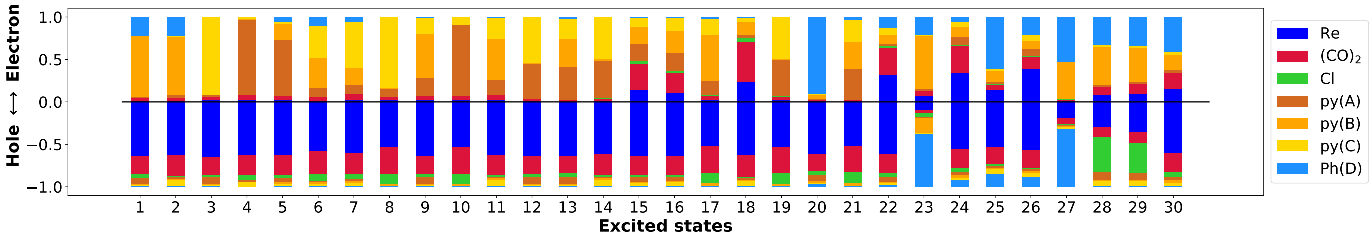

(b) Fragment decomposition of the hole/electron populations for each excited state.

Figure S22: Excited State Analysis of Complex **4a**

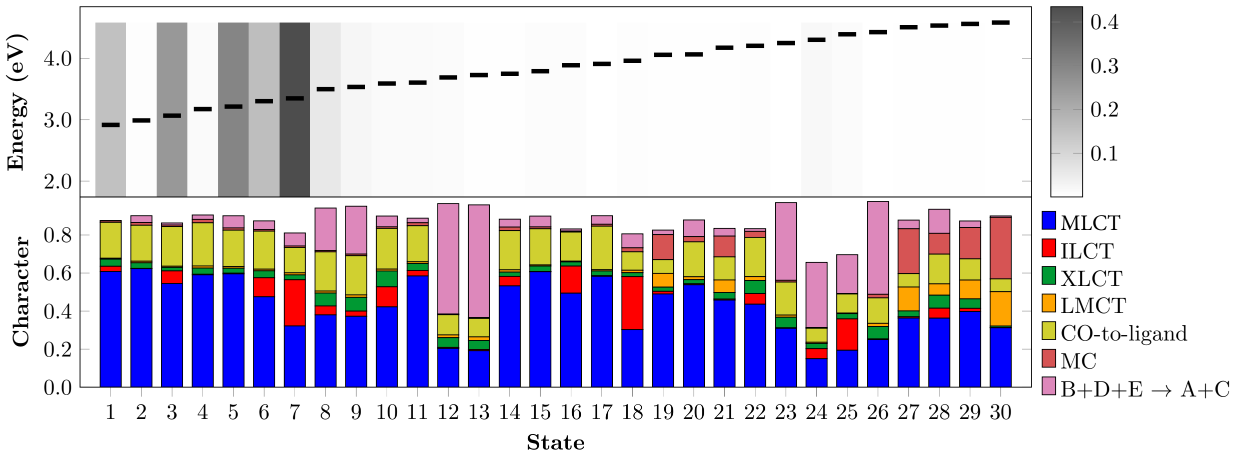

(a) Character decomposition [bottom row]; energies (horizontal lines) and oscillator strengths (as shading intensity) [top row].

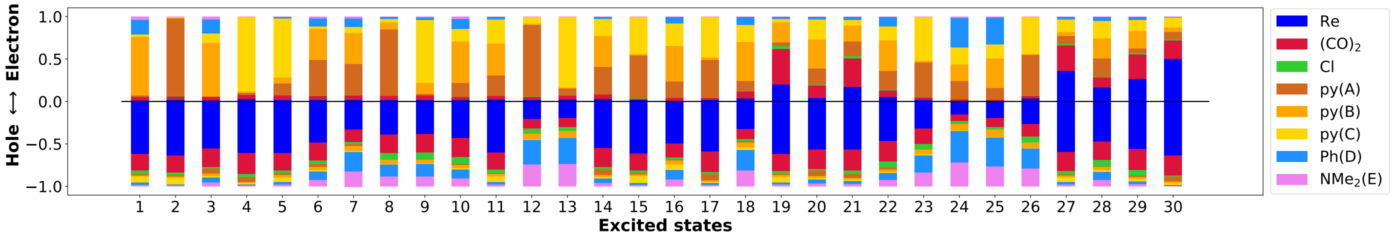

(b) Fragment decomposition of the hole/electron populations for each excited state.

Figure S23: Excited State Analysis of Complex **4b**

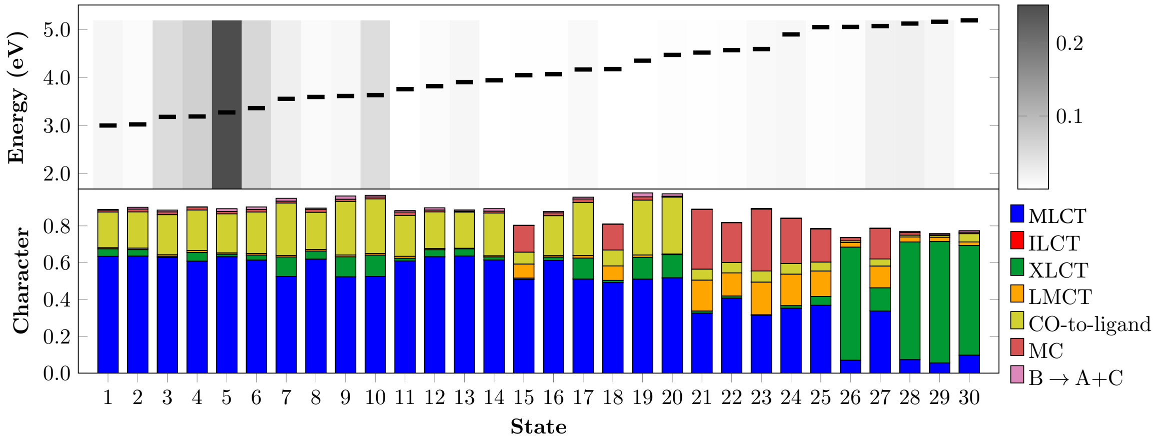

(a) Character decomposition [bottom row]; energies (horizontal lines) and oscillator strengths (as shading intensity) [top row].

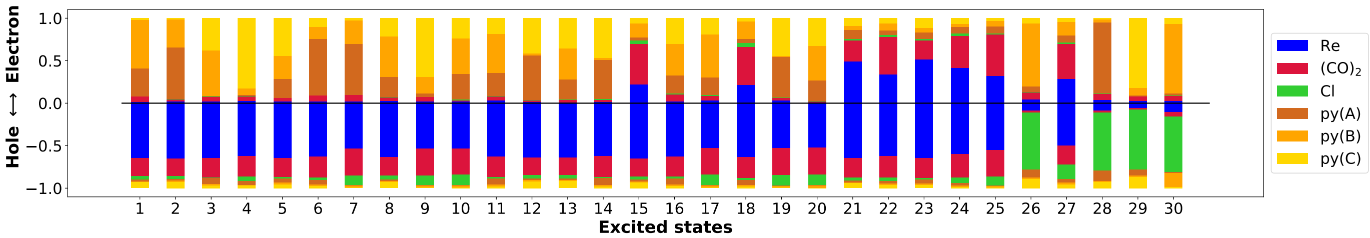

(b) Fragment decomposition of the hole/electron populations for each excited state.

Figure S24: Excited State Analysis of Complex **5a**

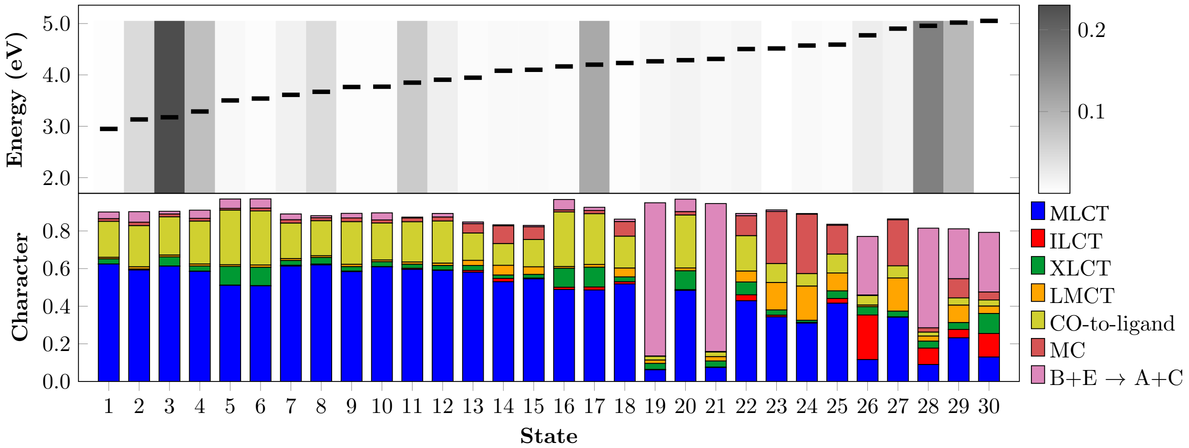

(a) Character decomposition [bottom row]; energies (horizontal lines) and oscillator strengths (as shading intensity) [top row].

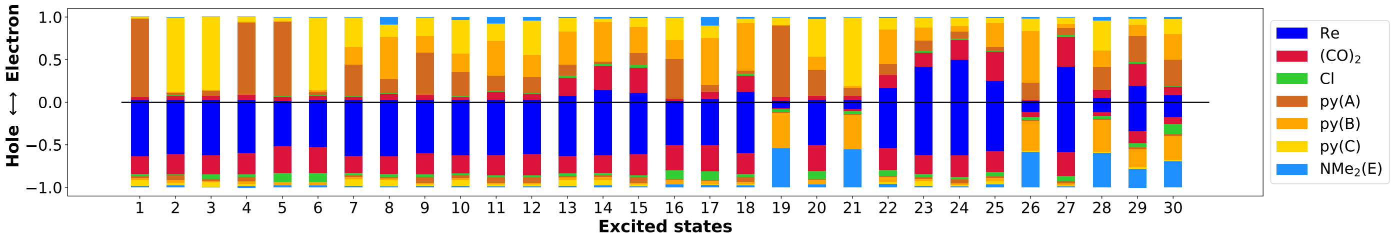

(b) Fragment decomposition of the hole/electron populations for each excited state.

Figure S25: Excited State Analysis of Complex **5b**

1.8 Charge Density Difference Isosurfaces

Charge density difference isosurfaces provide a graphical and intuitive picture of charge redistribution in the corresponding excited states. In all figures, blue corresponds to *less* electron density (i.e. where electron density comes from), and orange represents *increased* electron density (i.e. where electronic density goes to). The lowest 10 singlet states have energies below 4 eV ($\lambda > 310$ nm) and thus constitute the focus of the discussion in the manuscript.

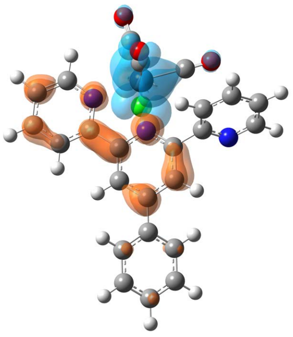

(a) $S_1 \leftarrow S_0$

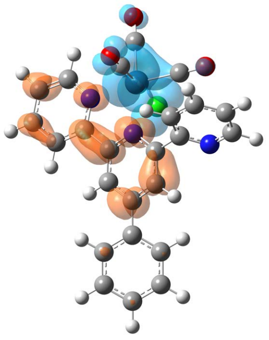

(b) $S_2 \leftarrow S_0$

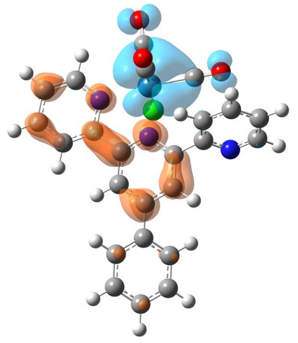

(c) $S_3 \leftarrow S_0$

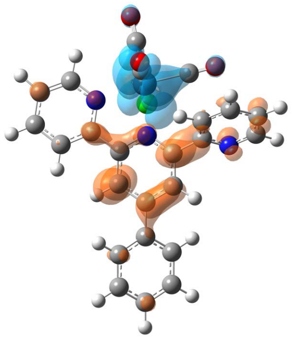

(d) $S_4 \leftarrow S_0$

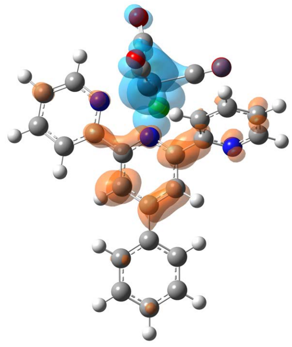

(e) $S_5 \leftarrow S_0$

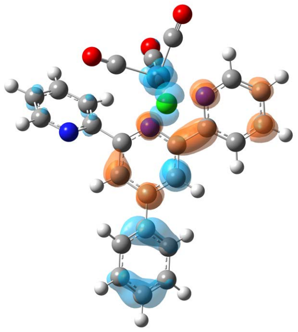

(f) $S_6 \leftarrow S_0$

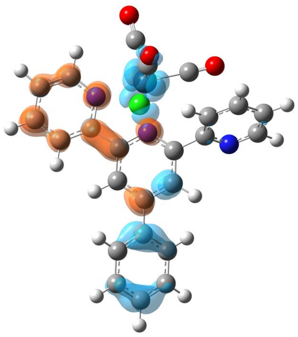

(g) $S_7 \leftarrow S_0$

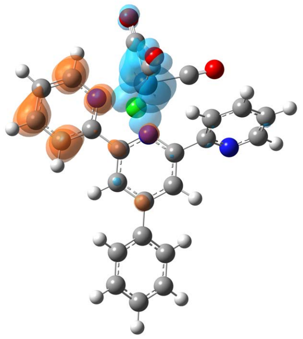

(h) $S_8 \leftarrow S_0$

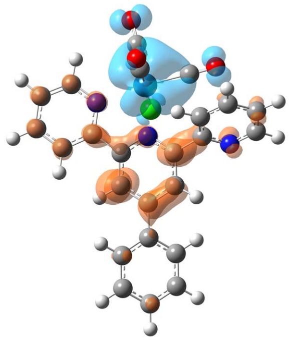

(i) $S_9 \leftarrow S_0$

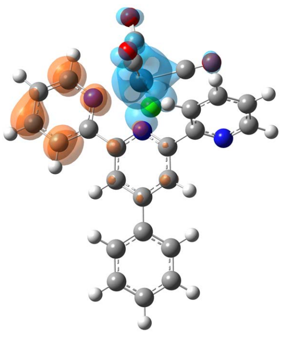

(j) $S_{10} \leftarrow S_0$

Figure S26: Charge Density Difference Isosurfaces of the lowest 10 singlets of **1a** at $|\Delta\rho| = 0.002$ a.u.

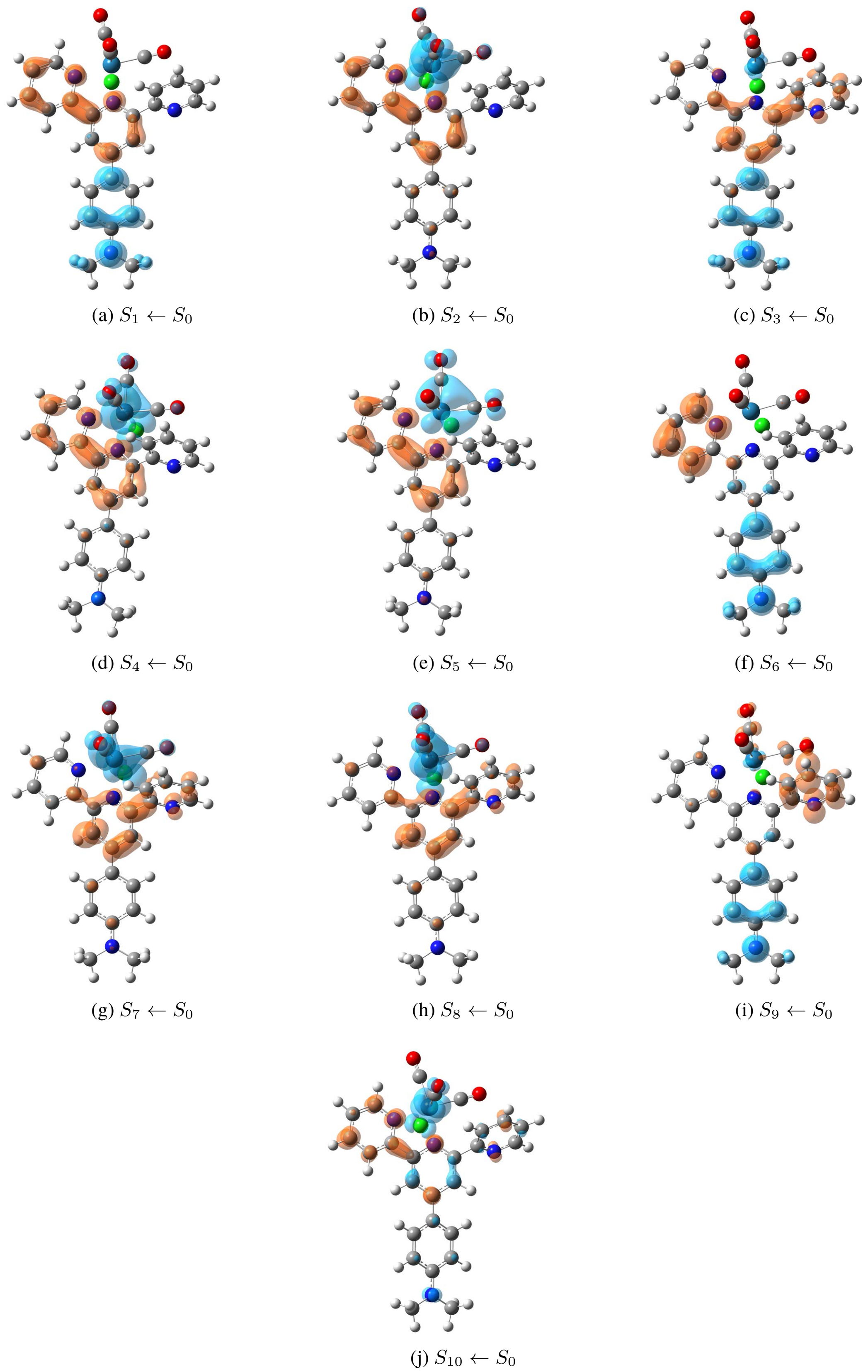

(a) $S_1 \leftarrow S_0$ (b) $S_2 \leftarrow S_0$ (c) $S_3 \leftarrow S_0$

(d) $S_4 \leftarrow S_0$ (e) $S_5 \leftarrow S_0$ (f) $S_6 \leftarrow S_0$

(g) $S_7 \leftarrow S_0$ (h) $S_8 \leftarrow S_0$ (i) $S_9 \leftarrow S_0$

(j) $S_{10} \leftarrow S_0$

Figure S27: Charge Density Difference Isosurfaces of the lowest 10 singlets of **1b** at $|\Delta\rho| = 0.002$ a.u.

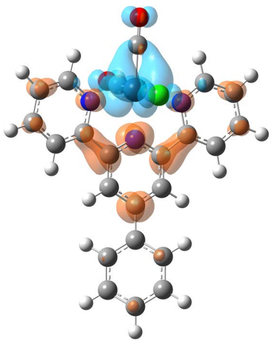

(a) $S_1 \leftarrow S_0$

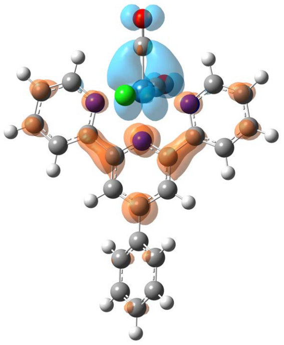

(b) $S_2 \leftarrow S_0$

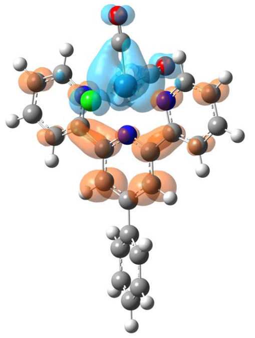

(c) $S_3 \leftarrow S_0$

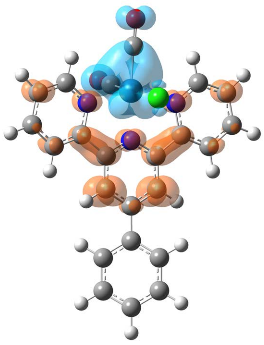

(d) $S_4 \leftarrow S_0$

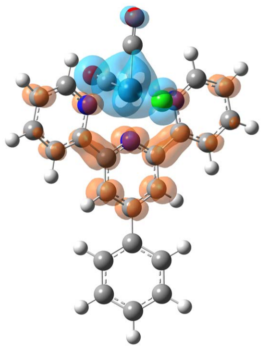

(e) $S_5 \leftarrow S_0$

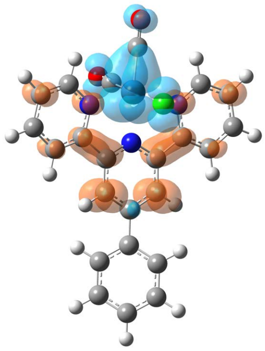

(f) $S_6 \leftarrow S_0$

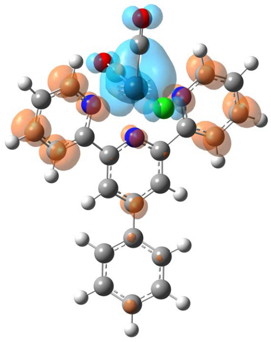

(g) $S_7 \leftarrow S_0$

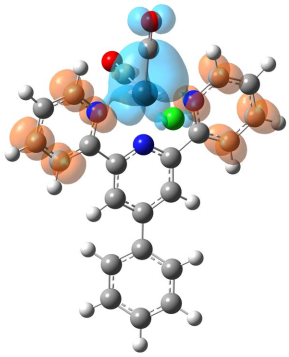

(h) $S_8 \leftarrow S_0$

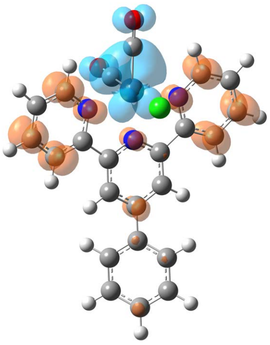

(i) $S_9 \leftarrow S_0$

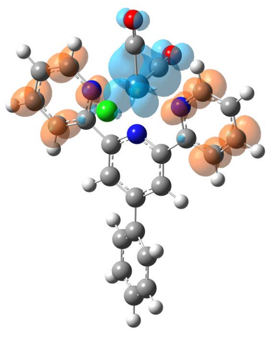

(j) $S_{10} \leftarrow S_0$

Figure S28: Charge Density Difference Isosurfaces of the lowest 10 singlets of **2a** at $|\Delta\rho| = 0.002$ a.u.

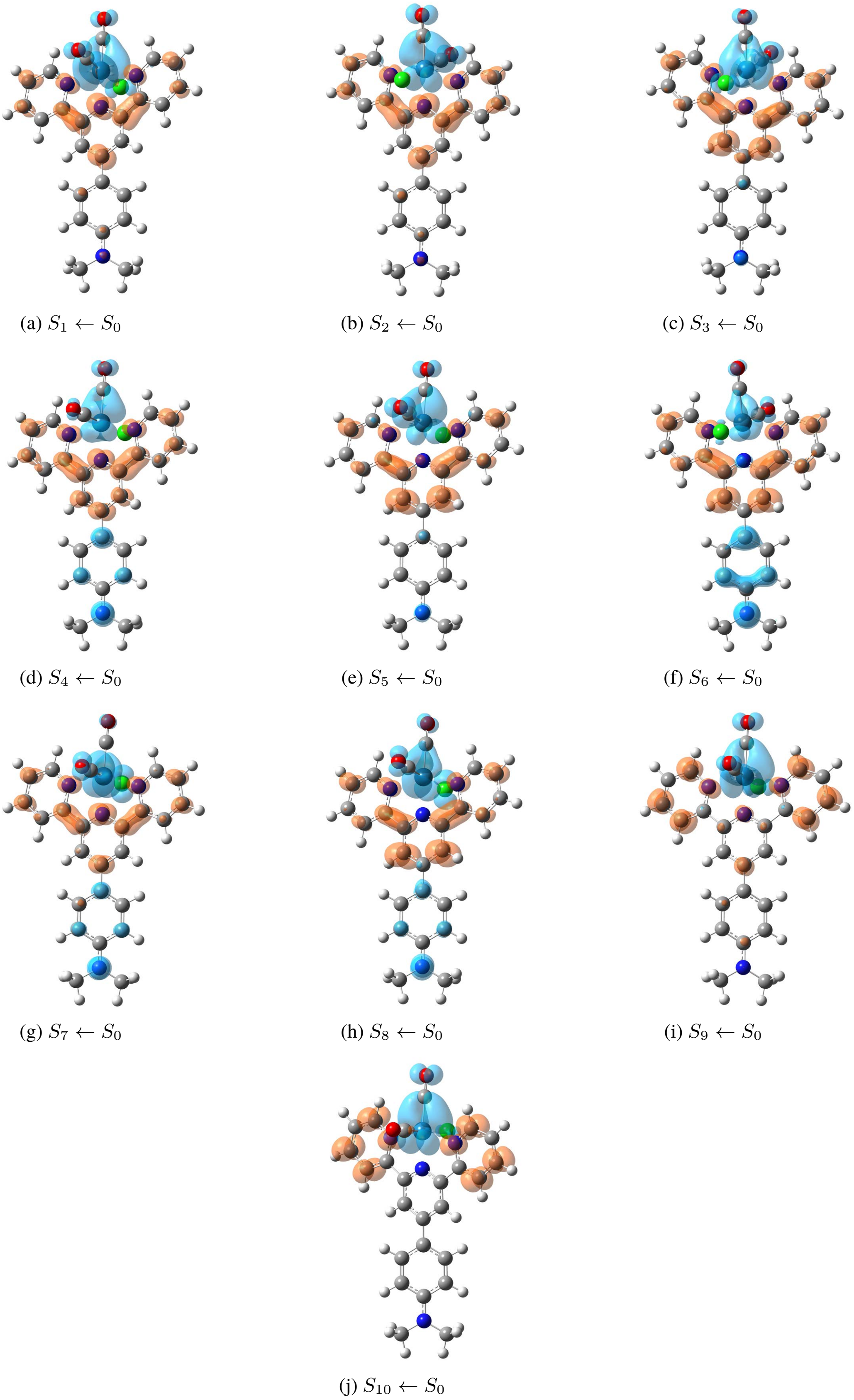

(a) $S_1 \leftarrow S_0$ (b) $S_2 \leftarrow S_0$ (c) $S_3 \leftarrow S_0$

(d) $S_4 \leftarrow S_0$ (e) $S_5 \leftarrow S_0$ (f) $S_6 \leftarrow S_0$

(g) $S_7 \leftarrow S_0$ (h) $S_8 \leftarrow S_0$ (i) $S_9 \leftarrow S_0$

(j) $S_{10} \leftarrow S_0$

Figure S29: Charge Density Difference Isosurfaces of the lowest 10 singlets of **2b** at $|\Delta\rho| = 0.002$ a.u.

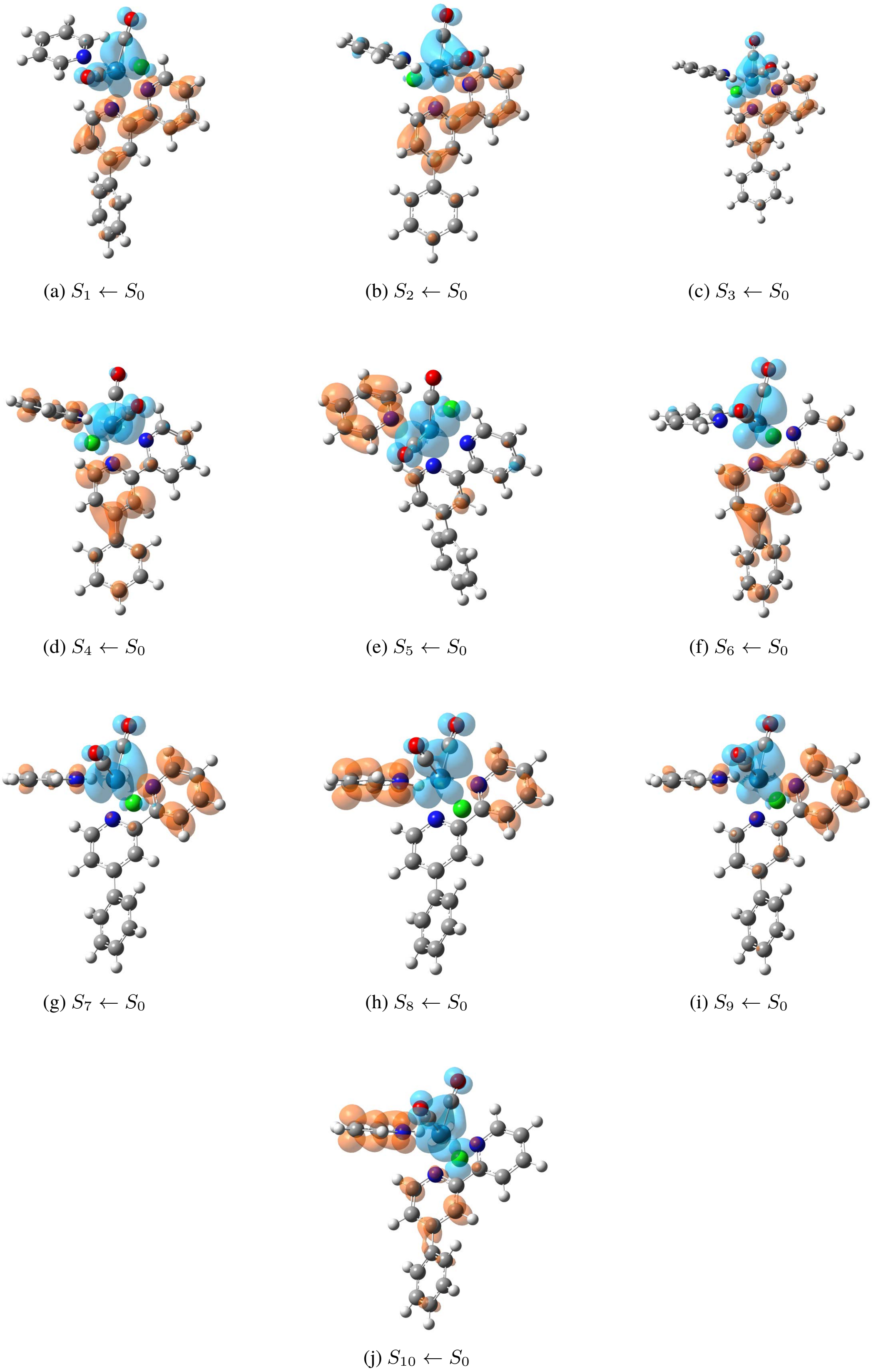

(a) $S_1 \leftarrow S_0$ (b) $S_2 \leftarrow S_0$ (c) $S_3 \leftarrow S_0$

(d) $S_4 \leftarrow S_0$ (e) $S_5 \leftarrow S_0$ (f) $S_6 \leftarrow S_0$

(g) $S_7 \leftarrow S_0$ (h) $S_8 \leftarrow S_0$ (i) $S_9 \leftarrow S_0$

(j) $S_{10} \leftarrow S_0$

Figure S30: Charge Density Difference Isosurfaces of the lowest 10 singlets of **3a** at $|\Delta\rho| = 0.002$ a.u.

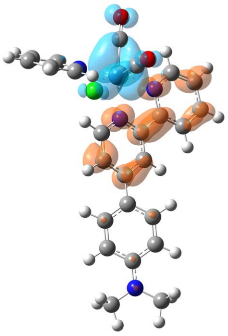

(a) $S_1 \leftarrow S_0$

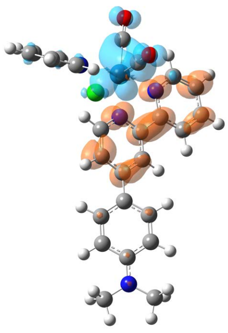

(b) $S_2 \leftarrow S_0$

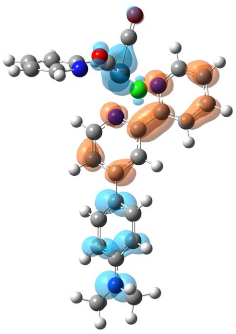

(c) $S_3 \leftarrow S_0$

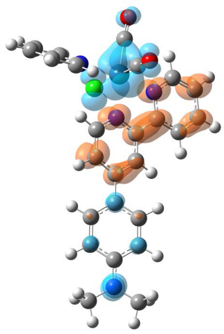

(d) $S_4 \leftarrow S_0$

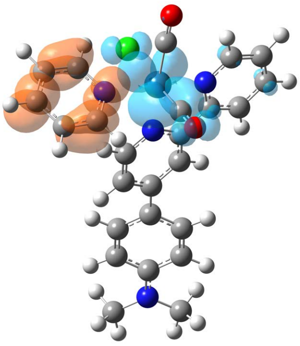

(e) $S_5 \leftarrow S_0$

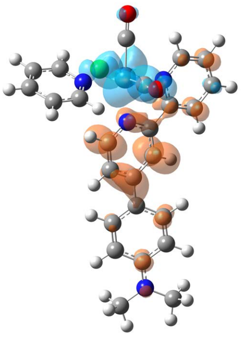

(f) $S_6 \leftarrow S_0$

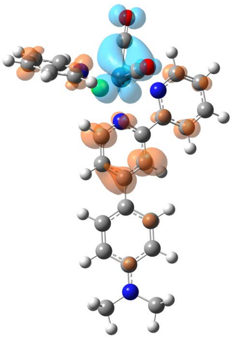

(g) $S_7 \leftarrow S_0$

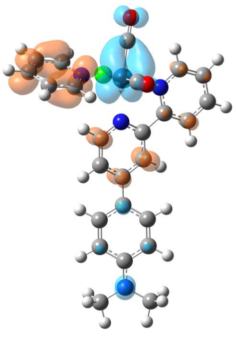

(h) $S_8 \leftarrow S_0$

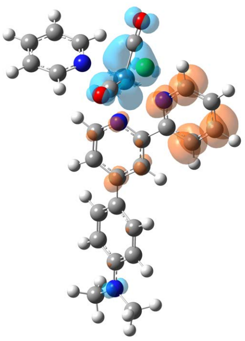

(i) $S_9 \leftarrow S_0$

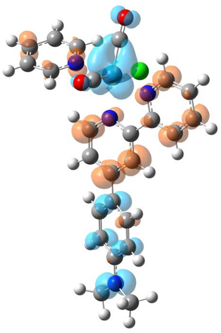

(j) $S_{10} \leftarrow S_0$

Figure S31: Charge Density Difference Isosurfaces of the lowest 10 singlets of **3b** at $|\Delta\rho| = 0.002$ a.u.

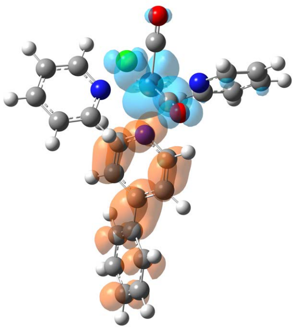

(a) $S_1 \leftarrow S_0$

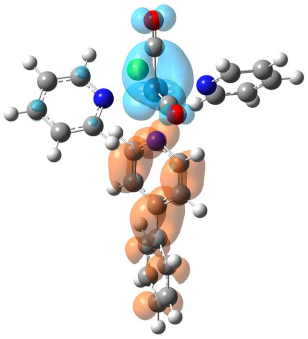

(b) $S_2 \leftarrow S_0$

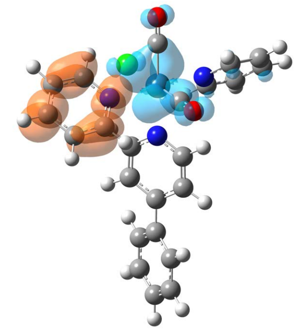

(c) $S_3 \leftarrow S_0$

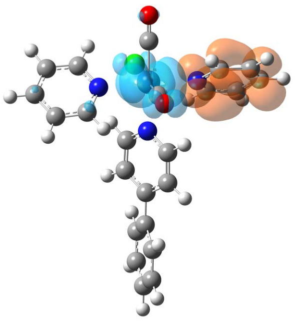

(d) $S_4 \leftarrow S_0$

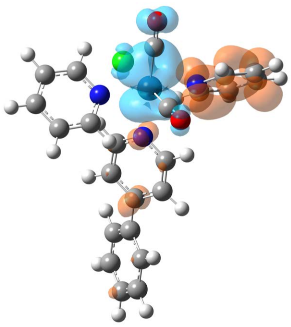

(e) $S_5 \leftarrow S_0$

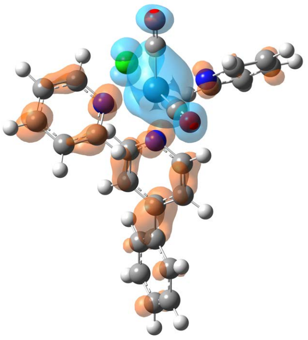

(f) $S_6 \leftarrow S_0$

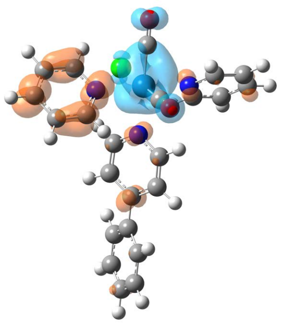

(g) $S_7 \leftarrow S_0$

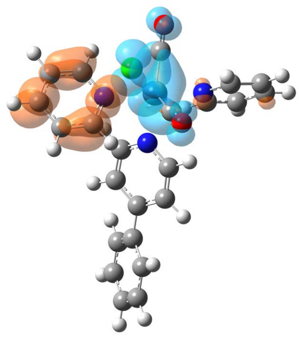

(h) $S_8 \leftarrow S_0$

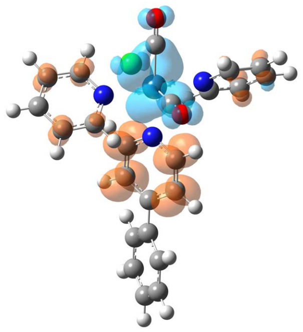

(i) $S_9 \leftarrow S_0$

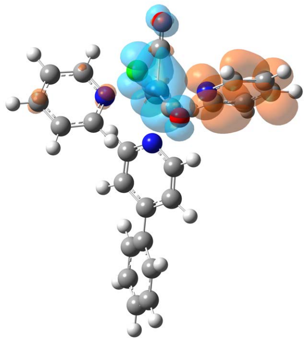

(j) $S_{10} \leftarrow S_0$

Figure S32: Charge Density Difference Isosurfaces of the lowest 10 singlets of **4a** at $|\Delta\rho| = 0.002$ a.u.

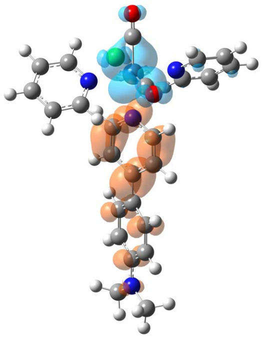

(a) $S_1 \leftarrow S_0$

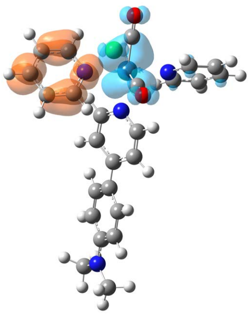

(b) $S_2 \leftarrow S_0$

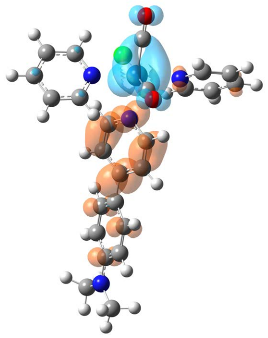

(c) $S_3 \leftarrow S_0$

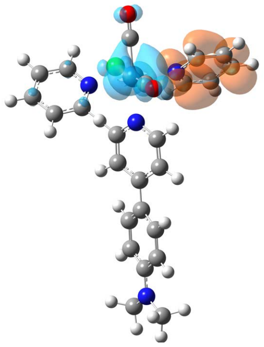

(d) $S_4 \leftarrow S_0$

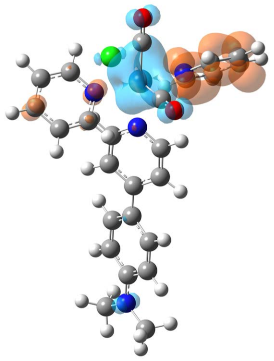

(e) $S_5 \leftarrow S_0$

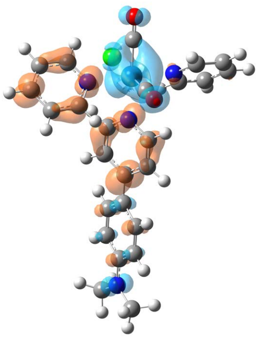

(f) $S_6 \leftarrow S_0$

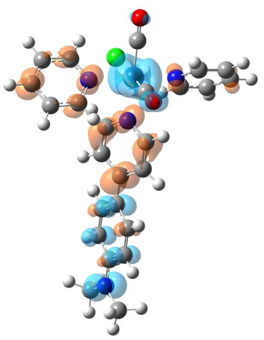

(g) $S_7 \leftarrow S_0$

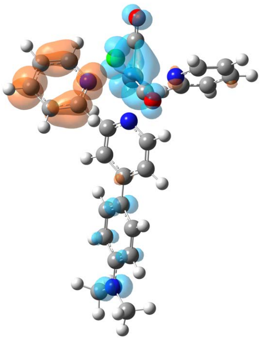

(h) $S_8 \leftarrow S_0$

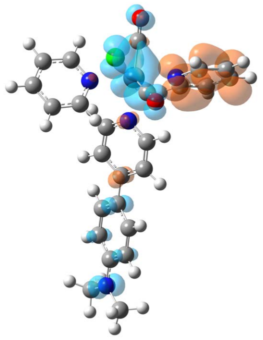

(i) $S_9 \leftarrow S_0$

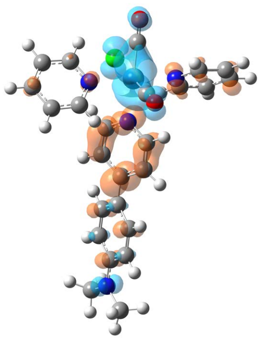

(j) $S_{10} \leftarrow S_0$

Figure S33: Charge Density Difference Isosurfaces of the lowest 10 singlets of **4b** at $|\Delta\rho| = 0.002$ a.u.

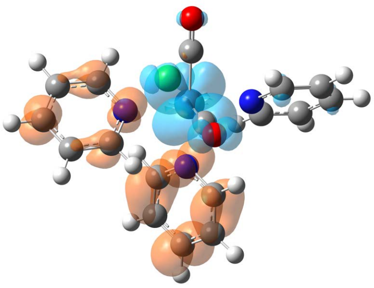

(a) $S_1 \leftarrow S_0$

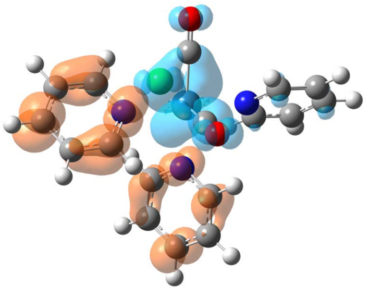

(b) $S_2 \leftarrow S_0$

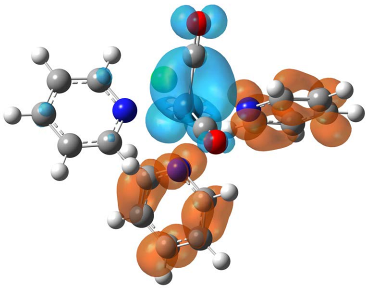

(c) $S_3 \leftarrow S_0$

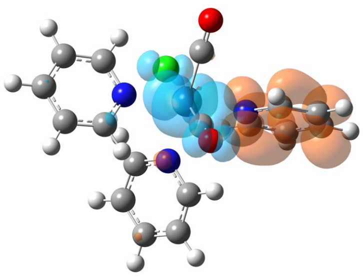

(d) $S_4 \leftarrow S_0$

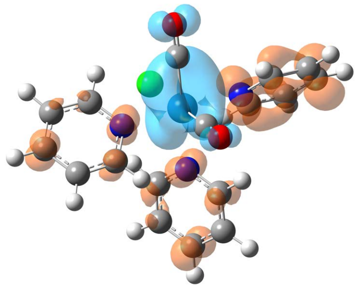

(e) $S_5 \leftarrow S_0$

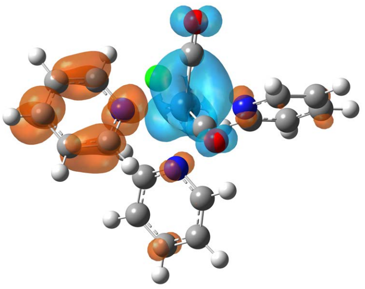

(f) $S_6 \leftarrow S_0$

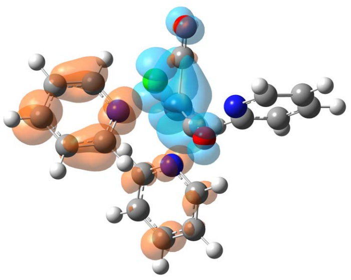

(g) $S_7 \leftarrow S_0$

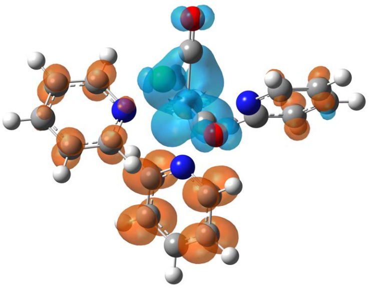

(h) $S_8 \leftarrow S_0$

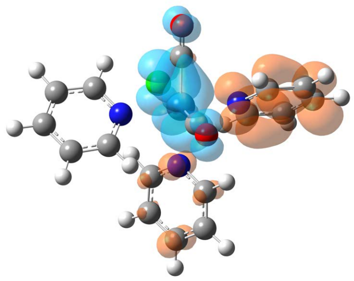

(i) $S_9 \leftarrow S_0$

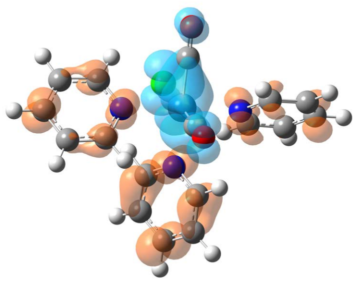

(j) $S_{10} \leftarrow S_0$

Figure S34: Charge Density Difference Isosurfaces of the lowest 10 singlets of **5a** at $|\Delta\rho| = 0.002$ a.u.

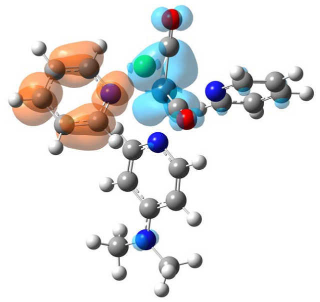

(a) $S_1 \leftarrow S_0$

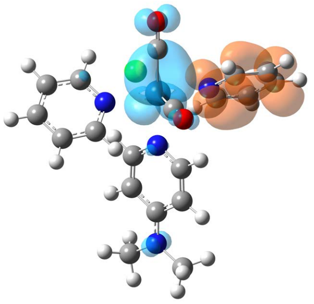

(b) $S_2 \leftarrow S_0$

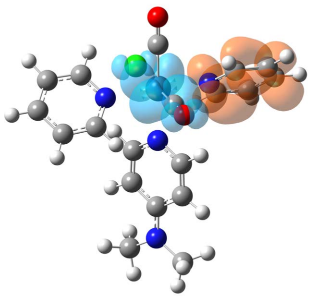

(c) $S_3 \leftarrow S_0$

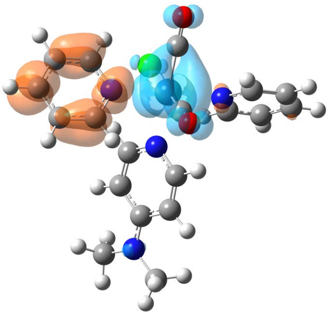

(d) $S_4 \leftarrow S_0$

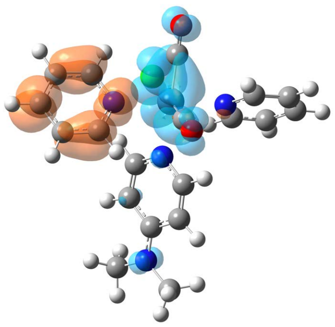

(e) $S_5 \leftarrow S_0$

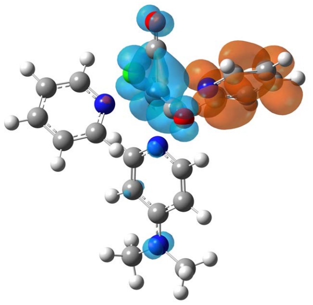

(f) $S_6 \leftarrow S_0$

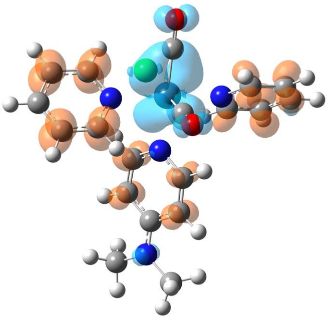

(g) $S_7 \leftarrow S_0$

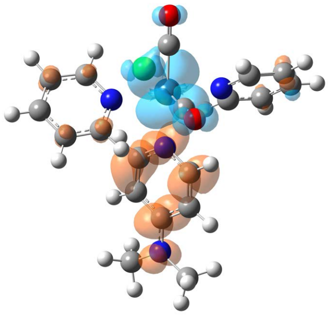

(h) $S_8 \leftarrow S_0$

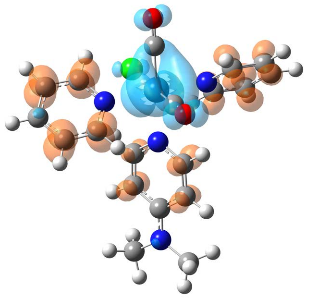

(i) $S_9 \leftarrow S_0$

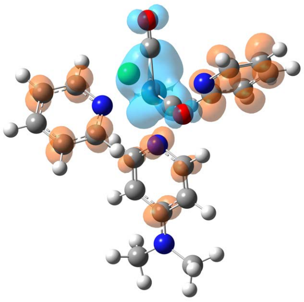

(j) $S_{10} \leftarrow S_0$

Figure S35: Charge Density Difference Isosurfaces of the lowest 10 singlets of **5b** at $|\Delta\rho| = 0.002$ a.u.

2. References

(1) R. Fernández-Terán and L. Séverý, Living Long and Prosperous: Productive Intraligand Charge-Transfer States from a Rhenium(I) Terpyridine Photosensitizer with Enhanced Light Absorption, Inorg. Chem. **60**, 1334 (2021).
(2) F. Plasser, TheoDORE: A toolbox for a detailed and automated analysis of electronic excited state computations, J. Chem. Phys. **152**, 084108 (2020).
(3) F. Plasser, M. Wormit, and A. Dreuw, New tools for the systematic analysis and visualization of electronic excitations. I. Formalism, J. Chem. Phys. **141**, 024106 (2014).
(4) F. Plasser and H. Lischka, Analysis of excitonic and charge transfer interactions from quantum chemical calculations, J. Chem. Theory Comput. **8**, 2777 (2012).
(5) N. M. O'Boyle, A. L. Tenderholt, and K. M. Langner, Cclib: A library for package-independent computational chemistry algorithms, J. Comput. Chem. **29**, 839 (2008).
